# Exploring the Major Barriers to Physical Activity in Persons With Multiple Sclerosis: Observational Longitudinal Study

**DOI:** 10.2196/52733

**Published:** 2024-03-18

**Authors:** Chloé Sieber, Christina Haag, Ashley Polhemus, Sarah R Haile, Ramona Sylvester, Jan Kool, Roman Gonzenbach, Viktor von Wyl

**Affiliations:** 1 Institute for Implementation Science in Health Care University of Zurich Zurich Switzerland; 2 Epidemiology, Biostatistics and Prevention Institute University of Zurich Zurich Switzerland; 3 Valens Rehabilitation Centre Valens Switzerland

**Keywords:** physical activity, barriers to physical activity, Barriers to Health Promoting Activities for Disabled Persons scale, BHADP scale, multiple sclerosis, Fitbit, wearable

## Abstract

**Background:**

Physical activity (PA) represents a low-cost and readily available means of mitigating multiple sclerosis (MS) symptoms and alleviating the disease course. Nevertheless, persons with MS engage in lower levels of PA than the general population.

**Objective:**

This study aims to enhance the understanding of the barriers to PA engagement in persons with MS and to evaluate the applicability of the Barriers to Health Promoting Activities for Disabled Persons (BHADP) scale for assessing barriers to PA in persons with MS, by comparing the BHADP score with self-reported outcomes of fatigue, depression, self-efficacy, and health-related quality of life, as well as sensor-measured PA.

**Methods:**

Study participants (n=45; median age 46, IQR 40-51 years; median Expanded Disability Status Scale score 4.5, IQR 3.5-6) were recruited among persons with MS attending inpatient neurorehabilitation. They wore a Fitbit Inspire HR (Fitbit Inc) throughout their stay at the rehabilitation clinic (phase 1; 2-4 wk) and for the 4 following weeks at home (phase 2; 4 wk). Sensor-based step counts and cumulative minutes in moderate to vigorous PA were computed for the last 7 days at the clinic and at home. On the basis of PA during the last 7 end-of-study days, we grouped the study participants as active (≥10,000 steps/d) and less active (<10,000 steps/d) to explore PA barriers compared with PA level. PA barriers were repeatedly assessed through the BHADP scale. We described the relevance of the 18 barriers of the BHADP scale assessed at the end of the study and quantified their correlations with the Spearman correlation test. We evaluated the associations of the BHADP score with end-of-study reported outcomes of fatigue, depression, self-efficacy, and health-related quality of life with multivariable regression models. We performed separate regression analyses to examine the association of the BHADP score with different sensor-measured outcomes of PA.

**Results:**

The less active group reported higher scores for the BHADP items *Feeling what I do doesn’t help*, *No one to help me*, and *Lack of support from family/friends*. The BHADP items *Not interested* in PA and *Impairment* were positively correlated. The BHADP score was positively associated with measures of fatigue and depression and negatively associated with self-efficacy and health-related quality of life. The BHADP score showed an inverse relationship with the level of PA measured but not when dichotomized according to the recommended PA level thresholds.

**Conclusions:**

The BHADP scale is a valid and well-adapted tool for persons with MS because it reflects common MS symptoms such as fatigue and depression, as well as self-efficacy and health-related quality of life. Moreover, decreases in PA levels are often related to increases in specific barriers in the lives of persons with MS and should hence be addressed jointly in health care management.

## Introduction

### Background

For decades, physical activity (PA) was believed to exacerbate multiple sclerosis (MS) symptoms such as fatigue [1]. It was only in the late 1990s that positive effects of PA for persons with MS were recognized [2]. In the context of MS, PA can ameliorate physical and cognitive functions of persons with MS, improve their health-related quality of life, and mitigate fatigue symptoms [3]. PA is recommended as symptomatic treatment in persons with MS, and emerging data even suggest disease-modifying or preventive effects of PA on MS [4,5]. Notwithstanding these findings, persons with MS are, on average, less active than the general population [6].

Recent World Health Organization guidelines recommend that adults with disabilities (aged ≥18 years) engage in 150 to 300 minutes of moderate PA or 75 to 150 minutes of vigorous PA per week [7]. For additional benefits, adults with disabilities should undertake muscle-strengthening activities at least 2 days per week and multicomponent PA focusing on functional balance and strength training at least 3 days per week. The World Health Organization does not provide an equivalent recommendation for the number of steps per day. Nevertheless, a threshold of 10,000 daily steps is commonly associated with an active lifestyle [8-10].

Activity sensors and Fitbit devices in particular have seen increasing adoption in MS research over the past years [11]; for example, such devices have been used to reduce sedentary behavior in persons with MS [12] or for remote monitoring of MS disability [13]. Despite the lower accuracy of Fitbit sensors at lower activity intensity [14] and slower walking speed [15-18], particularly relevant in the case of persons with MS, earlier studies have demonstrated the validity of Fitbit sensors in measuring step count [19-21]. These sensors enable individualized, passive, and inconspicuous monitoring of various metrics, including PA intensity and step counts, over an extended period of time [22,23].

In view of the numerous positive effects of PA on the health of persons with MS, it is crucial to understand facilitators as well as barriers to regular PA in general to achieve the recommended World Health Organization PA thresholds. However, understanding PA barriers can be challenging because they may be highly individual and multidimensional [24]. As for the latter, a narrative review identified at least five dimensions of PA barriers in persons with MS: (1) MS-related impairment and disability; (2) personal attitudes; (3) fatigue as a highly prevalent symptom; (4) the perceived benefits of exercise; and (5) logistical factors, including finances, support, and accessibility [25].

The multitude of possible influencing factors for PA levels makes studies on barriers to PA methodologically challenging. Among existing assessment frameworks for PA barriers, the Barriers to Health Promoting Activities for Disabled Persons (BHADP) scale plays a prominent role in studies concerning persons with MS [26]. However, research is lacking on whether the BHADP scale is a valid measure to understand PA barriers and their effects in real-world settings and to inform effective interventions to increase PA levels; for example, it remains unclear how the severity of PA barriers is perceived by active (≥10,000 steps/d) and less active (<10,000 steps/d) persons with MS, which social (eg, peer support) or health factors (eg, prevalent MS symptoms) may mitigate or exacerbate perceived barriers, and to what extent PA barriers decrease real-world PA.

### Objectives

Therefore, this analysis aimed to (1) compare PA barriers—as summarized by the BHADP scale—between physically active and less active persons with MS, (2) examine how other health factors such as fatigue or depression are independently associated with the BHADP score, and (3) explore the association of the BHADP score with sensor-measured outcomes of PA. Combined, these analyses contribute to the understanding of measurement characteristics and the validity of the BHADP scale in persons with MS.

## Methods

### Data Source

The data used in this study originated from the *Barrieren für körperliche Aktivität bei Multiple Sklerosis-Betroffenen* (BarKA-MS; Barriers to Physical Activity in People With Multiple Sclerosis) study, a 2-phased observational longitudinal cohort study repeatedly assessing barriers to PA and continuously measuring PA levels of persons with MS with a consumer-grade fitness tracker [27]. In the first phase (2-4 wk), persons with MS who were recruited at a rehabilitation clinic—Kliniken Valens, Switzerland—attended an inpatient rehabilitation program. The second phase corresponded to the first 4 weeks after the participants returned home. This analysis focuses on the primary objective of our trial preregistration.

### Ethical Considerations

The BarKA-MS study was approved by the ethics committee of the canton of Zurich (BASEC 2020-02350). All study participants provided written informed consent. Upon completion of the study, they were permitted to retain the consumer-grade fitness tracker used to measure PA during the study. No additional incentives were provided. The data was analyzed in a de-identified format.

### Eligibility and Recruitment

The BarKA-MS study aimed to recruit 45 participants. This target sample size was determined on the basis of similar studies [19], recent recommendations from the literature [28], and feasibility considerations. The feasibility considerations encompassed factors such as the number of potentially eligible persons with MS attending neurorehabilitation. All persons with MS attending an inpatient rehabilitation program at Kliniken Valens were considered eligible for inclusion in the study. The following eligibility criteria were considered for recruitment into the BarKA-MS study: (1) be aged ≥18 years; (2) present a confirmed diagnosis of MS (relapsing or progressive form); (3) have an Expanded Disability Status Scale (EDSS) score of 2.0 to 6.5 (ie, with reduced walking ability but still able to walk independently with or without an assistive device) and not use a wheelchair at home; (4) be able to complete the weekly questionnaires in German; (5) own a mobile device with Bluetooth functionality, such as a mobile phone or a tablet, required for the Fitbit synchronization; and (6) willingness to participate. Persons with MS who were unable to either (1) complete the baseline questionnaires or activate the Fitbit device or (2) adhere to the study procedures safely were deemed ineligible for participation. In addition, study participants who withdrew their informed consent were excluded from the study. Data collection was finalized in mid-November 2021. More details about the recruitment are provided elsewhere [29].

### Inpatient Rehabilitation Program

Throughout the inpatient rehabilitation program, study participants followed a personalized therapy plan, concentrating on individualized goals. Physiotherapy, which included balance and endurance training, was an important component of the rehabilitation program, with persons with MS attending 5 to 6 sessions per week, each lasting 30 to 60 minutes. In addition, study participants engaged in strength training 3 times per week, with each session lasting 30 to 45 minutes, and occupational therapy sessions 2 to 3 times per week for 30 minutes each, focusing on everyday life activities as well as arm and hand training. Furthermore, depending on the specific needs of the participants, other therapies were prescribed, including treadmills, water therapy, hippotherapy, and therapies that included virtual reality apps.

At the conclusion of inpatient rehabilitation, study participants were provided with an individualized training plan comprising 3 to 4 exercises to be performed at home. They were instructed on the proper execution of these exercises and received the instructions either in printed form or through an app, which included videos and photos based on the patient’s preferences. Caregivers offered encouragement in a relatively unstructured manner, encouraging participants to engage in these exercises at home and maintain PA.

### Variables

#### Measures

The BarKA-MS study participants were instructed to wear a Fitbit Inspire HR (Google LLC) during waking hours on their nondominant wrist throughout the study. The validity of the Fitbit Inspire HR–collected data in the context of our study was demonstrated previously [21]. Median step count and cumulative minutes in moderate to vigorous PA (MVPA) over the last 7 measurement days at the rehabilitation clinic and the last 7 measurement days at the end of the study (ie, 4 weeks after rehabilitation discharge) were used in the analyses (refer to Multimedia Appendix 1 [29-36] for more details about the Fitbit data processing). The sensor data were continuously collected using Fitabase (Small Steps Labs LLC), a secure commercial data aggregation platform for wearable devices.

Throughout the study, participants were invited to complete web-based questionnaires using the Research Management Information System survey platform [37]. At study enrollment, demographic (ie, sex, age, nationality, marital status, education, and employment status), and health (ie, MS type, MS duration, time since last relapse, and comorbidities) information were collected with the support of the recruiting on-site study coordinator. Additional measures such as BMI and EDSS score were assessed at study enrollment and at the end of the inpatient rehabilitation stay by medical professionals. Study participants also completed web-based patient-reported instruments, including the 12-item Multiple Sclerosis Walking Scale (range 0-100 [lowest walking ability]; refers to the last 2 weeks) [38], Fatigue Scale for Motor and Cognitive Functions (FSMC; range 20-100 [highest fatigue]; refers to everyday life) [39], General Self-Efficacy Scale (GSE; range 10-40 [highest self-efficacy]; refers to everyday life) [40], the 8-item Patient Health Questionnaire depression scale (PHQ-8; range 0-24 [severe depression]; refers to the last 2 weeks) [41], EQ-5D-5L (weighted using the French values set; range 0-100 [best quality of life]; refers to *today*) [42,43], and a visual analog scale to assess pain (“How bad was your pain when it was at its worst during the last 7 days?”; range 0-10 [worst pain]). The 12-item Multiple Sclerosis Walking Scale and the FSMC were developed for persons with MS and are well validated for this population [38,39]. By contrast, the GSE, PHQ-8, and EQ-5D-5L were not developed for persons with MS in the first place but were subsequently validated among this population group as well [40-44]. These patient-reported outcomes were recorded at enrollment, at the end of the inpatient rehabilitation stay, and at the end of the study. The main variable of interest was the BHADP score to measure barriers to PA. The BHADP scale, which was originally designed to evaluate the frequency of barriers to health promoting activities among individuals who are disabled, was additionally used for assessing the barriers to PA in persons with MS [26]. The BHADP scale comprises 18 items, scored from 1 to 4, leading to a total score of 18 to 72 points, with higher scores indicating greater PA barriers [26,45,46]. As the BHADP scale is only available in English, we translated it into German. A back translation into English confirmed the high consistency of both versions. The BHADP score was assessed at 3 time points of the BarKA-MS study: at study enrollment, at the end of the inpatient rehabilitation (2-4 weeks after enrollment, our analysis baseline), and at the end of the study (4 weeks after discharge). In addition, study participants were invited to answer the following free-text questions about PA engagement on a weekly basis. The first question pertained to the barriers to PA: “What kept you from being physically active this week?” The second question pertained to PA facilitators: “What made it easier for you to be physically active this week?” (refer to Multimedia Appendix 1 for more details). Further details on the BarKA-MS study, including measures that were not used for this analysis, are reported elsewhere [29].

#### Statistical Analysis

As part of study aim 1 (ie, the comparison of barriers to PA between active and less active persons with MS), descriptive statistics were used to characterize active and less active study participants. To this end, we considered participants *active* if the median daily step count over the last 7 valid wear days in home settings exceeded 10,000 steps; otherwise, the participants were assigned to the *less active* group [8]. For the group comparison, continuous variables were described as medians and IQRs and categorical variables as frequency counts and percentages. Furthermore, we described and compared the 18 barriers of the BHADP scale between the 2 activity groups by using unpaired 2-tailed *t* tests with Welch corrections for unequal variance.

For study aim 2 (ie, the examination of the association of health factors with the BHADP score), we examined the correlations among the 18 barriers of the BHADP scale assessed at the end of the study. In addition, we explored the construct validity, that is, the associations of the BHADP score with external criteria, which, in this case, are end-of-study reported outcomes of fatigue, depression, self-efficacy, and health-related quality of life. These analyses were based on Spearman correlations and unstandardized multivariable regression models. The multivariable regression models included the baseline variables age, sex, MS duration in years, and continuous forms of EDSS and BMI. The regression analyses were conducted on the imputed data set (refer to Multimedia Appendix 1 for more details).

In the context of study aim 3 (ie, the investigation of the association of the BHADP score with PA level), we conducted linear and logistic multivariable regression analyses to examine the association of the BHADP score assessed at the end of the study (explanatory variable) with sensor-based PA level (outcomes) measured over the last 7 end-of-study days. As sensor-based PA outcomes, we investigated median step counts and median cumulative minutes in MVPA in a continuous manner, as well as dichotomized median step counts (<10,000 or ≥10,000 steps/d) and dichotomized median cumulative minutes in MVPA (<150 or ≥150 min MVPA/wk). Basic multivariable regression models were controlled for the same baseline sociodemographic and health characteristics as in the regression analysis for aim 2. Further extensions of basic regression models were additionally controlled for either the PA level or the BHADP score measured at the end of rehabilitation, or both, to account for individualized starting levels at analysis baseline. As this is a mainly exploratory study, we did not correct for multiple testing. The regression analyses were conducted on the imputed data set. The results tables were presented using the *gtsummary* package (version 1.6.1) in R.

All analyses were conducted in R (version 4.2.1; R Foundation for Statistical Computing) [47], using the RStudio environment (version 2022.7.1.554; Posit Software, PBC) [48].

## Results

### Baseline Characteristics

Between January and September 2021, a total of 47 persons with MS were recruited during inpatient rehabilitation at Kliniken Valens to participate in the BarKA-MS study. Of the 47 participants, 2 (4%) withdrew from the study owing to reasons unrelated to either the study or their disease level [29]; thus, 45 (96%) participants completed the study. The characteristics of all study participants and participant subgroups based on their daily step count (<10,000 or ≥10,000 steps/d) are presented in Table 1. Of the 45 participants, 33 (73%) made up the less active subgroup, whereas 12 (27%) made up the active subgroup. Similar descriptive statistics were obtained in the sensitive analysis based on a threshold of 7000 steps per day (Table S1 in Multimedia Appendix 1).

During the last week of rehabilitation (analysis baseline), the 45 study participants performed, in median, 8656 (IQR 6103-10547) steps per day and 231 (IQR 86-478) minutes of MVPA per week. During the last week of the study at home (ie, 4 weeks after rehabilitation discharge), the participants accomplished, in median, 27% (2327/8656) fewer steps per day (ie, 6329/8656, 73% steps) and 51% (118/231) fewer minutes of MVPA per week (ie, 113/231, 49% min) than during the last week of rehabilitation (full distributions are shown in Figures S1-S4 in Multimedia Appendix 1).

**Table 1:** Study participants’ characteristics.

Characteristics				Study participants (n=45)		Less active study participants (<10,000 steps/d; n=33)	Active study participants (≥10,000 steps/d; n=12)
**Baseline demographics**							
	**Sex** **, n (%)**						
		Female	29 (64)		21 (64)		8 (67)
		Male	16 (36)		12 (36)		4 (33)
	Age (y), median (IQR)			46 (40-51)		48 (43-53)	44 (40-46)
	**Nationality^a^, n (%)**						
		Swiss	34 (76)		25 (76)		9 (75)
		German	6 (13)		5 (15)		1 (8)
		Italian	2 (4)		1 (3)		1 (8)
		Other	3 (7)		2 (6)		1 (8)
	**Marital status, n (%)**						
		Single	12 (27)		10 (30)		2 (17)
		Married	23 (51)		17 (52)		6 (50)
		Separated	1 (2)		1 (3)		N/A^b^
		Divorced	7 (16)		4 (12)		3 (25)
		Widowed	2 (4)		1 (3)		1 (8)
	**Education, n (%)**						
		Mandatory school not completed (or up to and including grade 7)	2 (4)		2 (6)		N/A
		Apprenticeship or secondary education completed (ie, *matura* schools or intermediate diploma schools)	25 (56)		18 (55)		7 (58)
		Higher professional education, universities of applied sciences, or university completed	18 (40)		13 (39)		5 (42)
	**Employment status, n (%)**						
		Working full time	5 (11)		4 (12)		1 (8)
		Working >50% but <100%	5 (11)		4 (12)		1 (8)
		Working ≤50%	17 (38)		12 (36)		5 (42)
		Not working	18 (40)		13 (39)		5 (42)
**Baseline health information**							
	**Multiple sclerosis type, n (%)**						
		Relapsing-remitting multiple sclerosis	18 (40)		11 (33)		7 (58)
		Primary-progressive multiple sclerosis	8 (18)		5 (15)		3 (25)
		Secondary-progressive multiple sclerosis	19 (42)		17 (52)		2 (17)
	Multiple sclerosis duration (y), median (IQR)			11 (5-21)		14 (5-23)	10 (3-12)
	Expanded Disability Status Scale score, median (IQR)			4.5 (3.5-6)		5 (3.5-6)	3.75 (2.9-4)
	**Expanded Disability Status Scale score, n (%)**						
		0-3.5	15 (33)		9 (27)		6 (50)
		4-5.5	18 (40)		13 (39)		5 (42)
		≥6	12 (27)		11 (33)		1 (8)
	**Time since last relapse (y)**						
		Value, median (IQR)	3 (1-5)		3 (1-6)		2 (1.5-4)
		Missing information, n (%)	8 (18)		7 (16)		1 (2)
	BMI (kg/m^2^), median (IQR)			24 (21-28)		23 (21-26)	27 (21-30.8)
	**BMI (kg/m^2^), n (%)**						
		<18.5 (underweight)	5 (11)		4 (12)		1 (8)
		18.5-24.9 (healthy weight)	22 (49)		18 (55)		4 (33)
		25.0-29.9 (overweight)	10 (22)		7 (21)		3 (25)
		≥30.0 (obesity)	8 (18)		4 (12)		4 (33)
	**Comorbidities^a^, n (%)**						
		None	18 (40)		13 (39)		5 (42)
		Hypertension	5 (11)		5 (15)		0 (0)
		Depression	5 (11)		5 (15)		0 (0)
		Skin diseases (eg, acne)	4 (9)		3 (9)		1 (8)
		Orthopedic diseases (eg, joint or back pain)	4 (9)		4 (12)		0 (0)
		Type 2 diabetes	3 (7)		2 (6)		1 (8)
		Migraine	2 (4)		N/A		2 (17)
		Hypothyroidism	2 (4)		1 (3)		1 (8)
		Other^c^	9 (20)		7 (21)		2 (17)
	**Change in the amount of sport practiced after the multiple sclerosis diagnosis, n (%)**						
		Less	27 (60)		21 (64)		6 (50)
		Same amount	2 (4)		1 (3)		1 (8)
		More	15 (33)		10 (30)		5 (42)
		Missing information	1 (2)		1 (3)		N/A
	Time spent at the rehabilitation clinic (d), median (IQR)			22 (18-26)		22 (18-26)	22 (19-24)
	Barriers to Health Promoting Activities for Disabled Persons scale score at analysis baseline (ie, at the end of the rehabilitation stay; range 18-72; the higher the score, the more barriers to physical activity), median (IQR)			20 (19-21)		20 (19-22)	20 (19-21)
**End-of-study assessments**							
	Barriers to Health Promoting Activities for Disabled Persons scale score (range 18-72; the higher the score, the more barriers to physical activity), median (IQR)			28 (24-35)		30 (24-35)	26 (25-28)
	**12-item Multiple Sclerosis Walking Scale score (range 0-100; the higher the score, the lower the walking ability)**						
		Value, median (IQR)	45.8 (29.2-79.2)		62.5 (35.4-85.4)		28.1 (16.1-29.2)
		Missing information, n (%)	6 (13)		4 (9)		2 (4)
	**Fatigue Scale for Motor and Cognitive Functions score (range 20-100; the higher the score, the more the fatigue), n (%)**						
		<43 (no fatigue)	9 (20)		7 (21)		2 (17)
		43-52 (mild fatigue)	6 (13)		5 (15)		1 (8)
		53-62 (moderate fatigue)	8 (18)		5 (15)		3 (25)
		≥63 (severe fatigue)	15 (33)		12 (36)		3 (25)
		Missing information	7 (16)		4 (12)		3 (25)
	**Fatigue Scale for Motor and Cognitive Functions–cognitive fatigue score (range 10-50; the higher the score, the more the fatigue), n (%)**						
		<22 (no cognitive fatigue)	17 (38)		14 (42)		3 (25)
		22-27 (mild cognitive fatigue)	6 (13)		4 (12)		2 (17)
		28-33 (moderate cognitive fatigue)	8 (18)		4 (12)		4 (33)
		≥34 (severe cognitive fatigue)	9 (20)		8 (24)		1 (8)
		Missing information	5 (11)		3 (9)		2 (17)
	**Fatigue Scale for Motor and Cognitive Functions–motor fatigue score (range 10-50; the higher the score, the more the fatigue), n (%)**						
		<22 (no motor fatigue)	6 (13)		5 (15)		1 (8)
		22-26 (mild motor fatigue)	4 (9)		2 (6)		2 (17)
		27-31 (moderate motor fatigue)	9 (20)		6 (18)		3 (25)
		≥32 (severe motor fatigue)	22 (49)		18 (55)		4 (33)
		Missing information	4 (9)		2 (6)		2 (17)
	General Self-Efficacy Scale score (range 10-40; the higher the score, the more the self-efficacy), median (IQR)			32 (30-36)		32 (29-36)	31 (30-36)
	**8-item Patient Health Questionnaire depression scale score (range 0-24; the higher the score, the more the depression signs), n (%)**						
		<10 (not clinically significant depression)	35 (78)		23 (70)		12 (100)
		≥10 (clinically significant depression)	7 (16)		7 (21)		0 (0)
		Missing information	3 (7)		3 (9)		0 (0)
	**EQ-5D-5L score, weighted by the French values set (range 0-100; the higher the score, the better the quality of life)**						
		Value, median (IQR)	63.5 (45.6-78.8)		63 (39.9-74.0)		78.3 (63.4-87.6)
		Missing information, n (%)	2 (4)		2 (6)		0 (0)
	“How bad was your pain when it was at its worst during the last 7 days?” (visual analog scale; range 0-10; the higher the score, the greater the pain), median (IQR)			3 (0-6)		3 (0-7)	3 (1-4)

^a^Multiple answers possible.

^b^N/A: not applicable.

^c^Asthma, type 1 diabetes, osteoporosis, psoriasis, cancer, rheumatic diseases, elevated cholesterol level, colitis ulcerosa, fibromyalgia, shingles, Meniere disease, and cerebellar syndrome.

### Description of Barriers to PA

**Figure 1:**
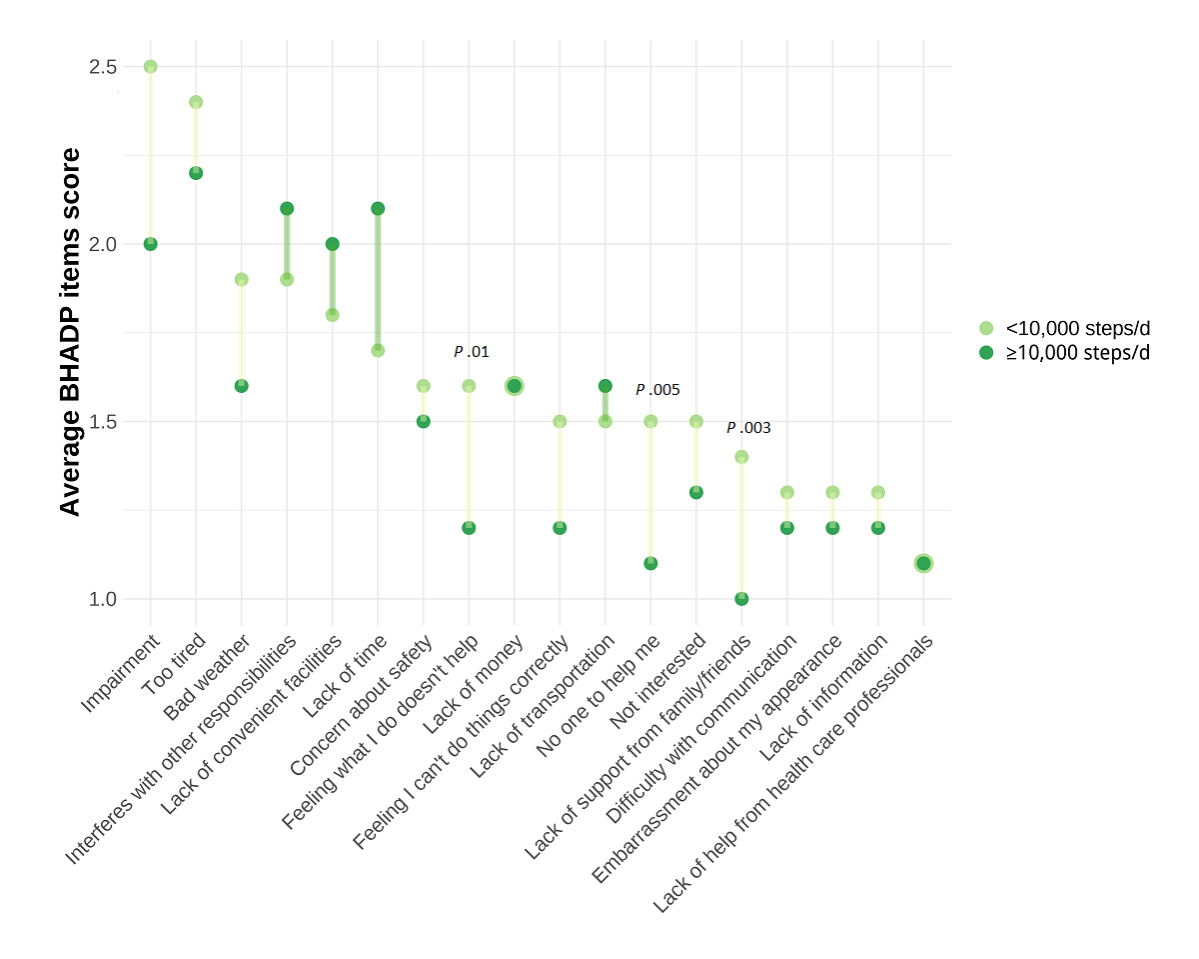
Barriers to physical activity by physical activity level. Average score of the 18 items of the Barriers to Health Promoting Activities for Disabled Persons (BHADP) scale (item score range 1-4) reported at the end of the study by the less active participants (<10,000 steps/d; n=33; in light green) and the active participants (≥10,000 steps/d; n=12; in dark green), in decreasing order for the less active participants. Statistically significant differences (*P*<.05) are reported directly on the graph. Higher scores reflect greater barriers. The figure is based on the complete cases data set.

Figure 1 illustrates the mean scores (on a range of 1-4) for the 18 BHADP items, stratified by participants’ PA level (means, SDs, *t* statistics, and *P* values are shown in Table S2 in Multimedia Appendix 1). The following items contained missing values, with the corresponding numbers provided in parentheses: *Lack of convenient facilities* (n=1), *Too tired* (n=2), *Lack of transportation* (n=1), *No one to help me* (n=1), *Concern about safety* (n=1), *Feeling I can’t do things correctly* (n=2), and *Difficulty with communication* (n=2). In both comparison groups, *Impairment* (mean 2.5, SD 1 for the less active group vs mean 2, SD 0.7 for the active group), *Too tired* (mean 2.4, SD 0.9 vs mean 2.2, SD 0.9), and *Interferes with other responsibilities* (mean 1.9, SD 0.9 vs mean 2.1, SD 0.9) were among the highest-rated barriers. The means and SDs at the study participants’ level are presented in Figure S5 and Table S3 in Multimedia Appendix 1. Most BHADP item scores were higher among the less active group. However, only a few exhibited statistical significance, which were *Feeling what I do doesn’t help* (mean 1.6, SD 0.7 for the less active group vs mean 1.2, SD 0.4 for the active group; *P*=.01), *No one to help me* (mean 1.5, SD 0.6 vs mean 1.1, SD 0.3; *P*=.005), and *Lack of support from family/friends* (mean 1.4, SD 0.7 vs mean 1, SD 0; *P*=.003). The *Impairment* item score was nominally higher in the less active group, but this difference was not statistically significant (mean 2.5, SD 1 vs mean 2.0, SD 0.7; *P*=.09). Similar results were observed in the sensitivity analysis based on a cutoff of <7000 or ≥7000 steps/d (Figure S6 in Multimedia Appendix 1). Furthermore, most of the BHADP item scores decreased at the end of the rehabilitation stay compared with before the rehabilitation stay (Figures S7 in Multimedia Appendix 1). However, at the end of the study (ie, at the end of the home phase), they rebounded to the start-of-rehabilitation levels (Figures S7-S9 in Multimedia Appendix 1). The items *Impairment* and *Too tired* improved significantly from study enrollment to the end of the study (*Impairment*: mean 2.9, SD 0.9 at study enrollment vs mean 2.4, SD 0.9 at the end of the study; *P*<.001; *Too tired*: mean 2.6, SD 1 at study enrollment vs mean 2.3, SD 0.9 at the end of the study; *P*=.04).

Barriers and facilitators to PA were additionally surveyed through weekly free-text questions (Figures S10 and S11 in Multimedia Appendix 1). The most frequently reported key words were *work*, *fatigue*, and *weather* (≥15 occurrences) in the question about PA barriers and *weather* and *motivation* (20 occurrences) in the question concerning the PA facilitators.

### Associations of Barriers Score

For study aim 2, we intended to examine the correlations among the 18 BHADP items, as well as the associations of the total BHADP score with other patient-reported instruments. The 18 items of the BHADP scale revealed interdependencies among different items (Figure S12 in Multimedia Appendix 1); for instance, *Not interested* in PA was positively correlated with *Impairment* (ρ=0.56; *P*=.02), *Difficulty with communication* (ρ=0.44; *P*=.04), and *Bad weather* (ρ=0.44; *P*=.01). The item *Bad weather* was also negatively correlated with *Interferes with other responsibilities* (ρ=−0.15; *P*=.02). Furthermore, the item *Interferes with other responsibilities* was positively associated with *Lack of time* (ρ=0.6; *P*<.001).

Moreover, given the high importance of the BHADP item *Impairment*, we further explored the associations of the overall BHADP score with specific patient-reported outcomes of fatigue, depression, self-efficacy, and health-related quality of life (Figure S13 in Multimedia Appendix 1). In particular, the total FSMC fatigue score (ρ=0.66; *P*=.002) and the PHQ-8 score for depression (ρ=0.73; *P*<.001) demonstrated a positive correlation with the BHADP score. The EQ-5D-5L score for health-related quality of life (ρ=−0.60; *P*<.001) and the GSE self-efficacy score (ρ=−0.67; *P*<.001) exhibited a negative correlation with the BHADP score. Multivariable, confounder-adjusted regression analyses (Table 2) confirmed the positive relationships of the PHQ-8 (β coefficient=0.90, 95% CI 0.56-1.2) and FSMC (β coefficient=0.16, 95% CI 0.07-0.25) scores with the BHADP score. In other words, an elevated depressive state and increased fatigue were independently associated with an increase in the barriers to PA. Similarly, the adjusted regression analyses substantiated the negative relationships of the EQ-5D-5L (β coefficient=−17, 95% CI −23 to −11) and GSE (β coefficient=−0.49, 95% CI −0.72 to −0.25) scores with the BHADP score. This suggests that higher health-related quality of life and increased self-efficacy are independently associated with a reduction in the barriers to PA. The regression models were re-estimated on the complete cases data set as a sensitivity analysis (Table S4 in Multimedia Appendix 1), which did not change the results substantially.

**Table 2:** Linear regression analyses with the Barriers to Health Promoting Activities for Disabled Persons (BHADP) scale score as outcome. Confounder-adjusted unstandardized linear regression models to assess the association of the BHADP score (dependent variable) with the 8-item Patient Health Questionnaire depression scale (PHQ-8), Fatigue Scale for Motor and Cognitive Functions (FSMC), EQ-5D-5L, and General Self-Efficacy Scale (GSE) scores (independent variables), based on the imputed data set (n=45). Notably, as the β coefficients were not standardized, they are not directly comparable across the different regression analyses.

Characteristic		BHADP score vs PHQ-8 score			BHADP score vs FSMC score			BHADP score vs EQ-5D-5L score			BHADP score vs GSE score	
		β coefficient (95% CI)	*P* value	β coefficient (95% CI)		*P* value	β coefficient (95% CI)		*P* value	β coefficient (95% CI)		*P* value
Intercept		29 (19 to 39)	<.001	26 (13 to 39)		<.001	54 (43 to 65)		<.001	50 (38 to 62)		<.001
Age		−0.07 (−0.25 to 11)	.40	−0.01 (−0.22 to 0.19)		.90	−0.02 (−0.20 to 0.16)		.80	−0.01 (−0.20 to 0.19)		.93
**Sex**												
	Female	—^a^	N/A^b^	—		N/A	—		N/A	—		N/A
	Male	0.96 (−2.2 to 4.1)	.50	1.7 (−1.8 to 5.2)		.30	1.3 (−1.7 to 4.3)		.40	2.2 (−1.1 to 5.5)		.20
BMI		−0.13 (−0.39 to 0.13)	.30	−0.23 (−0.52 to 0.06)		.11	−0.34 (−0.59 to −0.09)		.008	−0.16 (−0.45 to 0.12)		.20
MS^c^ duration		−0.12 (−0.28 to 0.04)	.14	−0.12 (−0.30 to 0.07)		.20	−0.15 (−0.31 to 0.01)		.06	−0.13 (−0.31 to 0.05)		.15
EDSS^d^ score		0.52 (−0.65 to 1.7)	.40	0.18 (−1.1 to 1.5)		.80	−0.95 (−2.1 to 0.23)		.11	−0.18 (−1.4 to 1.1)		.80
PHQ-8 score		0.90 (0.56 to 1.2)	<.001	N/A		N/A	N/A		N/A	N/A		N/A
FSMC score		N/A	N/A	0.16 (0.07 to 0.25)		<.001	N/A		N/A	N/A		N/A
EQ-5D-5L score		N/A	N/A	N/A		N/A	−17 (−23 to −11)		<.001	N/A		N/A
GSE score		N/A	N/A	N/A		N/A	N/A		N/A	−0.49 (−0.72 to −0.25)		<.001

^a^Reference category.

^b^N/A: not applicable.

^c^MS: multiple sclerosis.

^d^EDSS: Expanded Disability Status Scale.

Furthermore, we evaluated the relationships between 4 different PA outcome measures and the BHADP score by means of univariate and multivariable linear and logistic regressions (Table 3). The multivariable regressions were adjusted for the confounding variables age, sex, MS duration in years, and continuous forms of EDSS and BMI, assessed at baseline (regression details not shown). Overall, the dichotomized median step counts outcome (<10,000 or ≥10,000 steps/d; models 1, 2, and 3) and the dichotomized median cumulative minutes in MVPA outcome (<150 or ≥150 min MVPA/wk; models 7, 8, and 9) did not reveal statistically significant relationships with the total BHADP score. Similar results were observed in sensitivity analyses using a dichotomized median step counts outcome based on a cutoff of <7000 or ≥7000 steps per day (Table S5 in Multimedia Appendix 1). By contrast, the continuous outcomes median step counts and median cumulative minutes in MVPA exhibited statistically significant relationships with the BHADP score but only after additional adjustment for analysis baseline (ie, end of rehabilitation) step count (models 5 and 6) and MVPA levels (models 11 and 12), respectively. This suggests that an increase in median daily step counts and in median weekly cumulative minutes in MVPA were independently associated with a reduction in the barriers to PA. Specifically, a 1-unit increase in the BHADP score was associated with 218.84 (95% CI 50.86-386.82; model 5) and 210.27 (95% CI 39-381.54; model 6) fewer steps per day. Likewise, a 1-unit increase in BHADP score was associated with 15.04 (95% CI 1.1-28.99) and 14.41 (95% CI 0.1-28.72) fewer weekly MVPA minutes. Sensitivity analyses based on complete cases (Table S6 in Multimedia Appendix 1) and on PA data collected during the penultimate study week instead of the last study week (imputed and complete cases data; Table S7 in Multimedia Appendix 1) resulted in very similar findings, except that the continuous outcome–based linear regression analyses for weekly cumulative MVPA minutes did not exhibit statistically significant relationships with the BHADP score. Moreover, sensitivity analyses based on PA data collected during the penultimate study week revealed a lower decrease in the step count per day per 1-unit increase in the BHADP score. In the case of the imputed data, a 1-unit increase in the BHADP score was associated with 196.01 (95% CI 38.74-353.27; model 5) and 190.09 (95% CI 29.26-350.91; model 6) fewer steps per day.

**Table 3:** Imputed linear regressions with physical activity as outcome. Univariate and confounder-adjusted (ie, age, sex, multiple sclerosis duration in years, and continuous forms of Expanded Disability Status Scale and BMI assessed at baseline) multivariable regression models to evaluate the association of physical activity assessed during the last week of the study with the Barriers to Health Promoting Activities for Disabled Persons scale assessed at the end of the study, based on the imputed data set (n=45).

Models				Univariate imputed data analysis		Multivariable imputed data analysis^a^
**Last week of the study**						
	**1. ≥10,000 steps/d^b^**					
		Odds ratio (95% CI)	0.93 (0.82 to 1.06)		0.88 (0.74 to 1.04)	
		*P* value	.29		.14	
	**2. ≥10,000 steps/d controlled for steps/d at the end of the rehabilitation**					
		Odds ratio (95% CI)	0.86 (0.73 to 1.02)		0.82 (0.67 to 1.00)	
		*P* value	.09		.05	
	**3. ≥10,000 steps/d controlled for steps/d and barriers score at the end of the rehabilitation**					
		Odds ratio (95% CI; *P* value)	0.87 (0.71 to 1.05)		0.86 (0.69 to 1.06)	
		*P* value	.14		.15	
	**4. Steps/d**					
		β coefficient (95% CI)	−48.32 (−259.08 to 162.44)		−69.43 (−275.33 to 136.47)	
		*P* value	.65		.50	
	**5. Steps/d controlled for steps/d at the end of the rehabilitation**					
		β coefficient (95% CI)	−*164.28 (−321.17 to −7.38)*^c^		−*218.84 (−386.82 to −50.86)*	
		*P* value	*.04*		*.01*	
	**6. Steps/d controlled for steps/d and barriers score at the end of the rehabilitation**					
		β coefficient (95% CI)	−151.92 (−307.87 to 4.04)		−*210.27 (−381.54 to −39.00)*	
		*P* value	.06		*.02*	
	**7. ≥150 min of MVPA^d^/wk^e^**					
		Odds ratio (95% CI)	0.97 (0.87 to 1.08)		0.97 (0.86 to 1.11)	
		*P* value	.59		.67	
	**8. ≥150 min of MVPA/wk controlled for min of MVPA/wk at the end of the rehabilitation**					
		Odds ratio (95% CI)	0.94 (0.82 to 1.07)		0.95 (0.81 to 1.12)	
		*P* value	.34		.52	
	**9. ≥150 min of MVPA/wk controlled for min of MVPA/wk and barriers score at the end of the rehabilitation**					
		Odds ratio (95% CI)	0.95 (0.82 to 1.09)		0.95 (0.81 to 1.13)	
		*P* value	.44		.58	
	**10. Min of MVPA/wk**					
		β coefficient (95% CI)	−8.67 (−24.07 to 6.72)		−12.19 (−27.28 to 2.9)	
		*P* value	.26		.11	
	**11. Min of MVPA/wk controlled for min of MVPA/wk at the end of the rehabilitation**					
		β coefficient (95% CI)	−11.64 (−24.92 to 1.65)		−*15.04 (−28.99 to −1.1)*	
		*P* value	.08		*.04*	
	**12. Min of MVPA/wk controlled for min of MVPA/wk and barriers score at the end of the rehabilitation**					
		β coefficient (95% CI)	−10.85 (−24.26 to 2.56)		−*14.41 (−28.72 to −0.1)*	
		*P* value	.11		*.048*	

^a^Adjusted for age, sex, BMI, multiple sclerosis duration, and Expanded Disability Status Scale.

^b^Steps/d corresponds to the mean number of steps per day and per individual.

^c^Statistically significant effect sizes (*P*<.05) are marked in italics.

^d^MVPA: moderate to vigorous physical activity.

^e^Min of MVPA/wk corresponds to the sum of minutes of MVPA during the week.

## Discussion

### Principal Findings

We found that persons with MS with different levels of PA do not face the same barriers to engage in PA. Less active persons with MS express a greater need for general as well as family and friends’ support and empowerment to engage in PA. We tested the construct validity of the BHADP scale and found it suitable for use in persons with MS. In addition to evaluating barriers to PA, the scale reflects common MS symptoms such as fatigue and depression, as well as self-efficacy and health-related quality of life. Moreover, an increase in sensor-measured PA level was associated with a decrease in barriers to PA.

### Comparison With Prior Work

Overall, our findings are well aligned with previous studies. On the basis of longitudinal electronic surveys and Fitbit measurements in 45 participants, this study investigated the validity and usefulness of the BHADP score to explain real-world PA barriers and their consequences for sensor-measured PA among persons with MS.

We observed that less active persons with MS (<10,000 sensor-measured steps/d) were more likely to have signs of a more advanced disease stage, including a longer MS history, a higher EDSS score, and a higher proportion of secondary-progressive MS cases. Consistently, a recent Australian study observed lower PA levels among persons with MS with more severe symptoms [49]. The less active group also reported higher fatigue levels, as indicated by the FSMC score. This finding is consistent with a recent study that observed an association between increased fatigue and decreased PA [50]. Although many MS-related symptoms and impairments are only minimally modifiable, fatigue can be mitigated to some extent by pharmacological and nonpharmacological measures; for example, in disease management programs, persons with MS learn strategies to better manage their fatigue by adapting their daily routines to match the pattern of their fatigue [51]. Persons with MS can also gain a sense of empowerment through coaching and become better able to exert control over their energy levels [51]. PA can also positively influence fatigue [3] and health-related quality of life [52] once initial fatigue barriers have been overcome. Along similar lines, a subset of participants (7/33, 21%) in the less active group exhibited high PHQ-8 scores that are suggestive of severe depression, whereas none in the active group did. Most likely, this finding suggests that persons with depressive symptoms may struggle more often to be physically active. Nonetheless, several meta-analyses provided initial evidence that PA has the potential to decrease depression symptoms in persons with MS [53-56].

Furthermore, we found that the BHADP items *Not interested* in PA and *Impairment* were positively correlated—a noteworthy finding from a care management perspective. Impairments may reduce motivation for PA, which further decreases engagement in PA and leads to a vicious cycle [50]. The important effect of MS-related symptoms as PA barriers was further underscored in a multivariable regression analysis of validated patient-reported outcomes for fatigue, depression, lack of self-efficacy, and health-related quality of life on the BHADP score.

Moreover, the less active group reported not being sufficiently helped by their families and friends, whereas the active group generally did not cite a lack of assistance as a major barrier. These observations are corroborated by another study, which highlighted a positive relationship between the amount of support from relatives and the level of PA [57].

Study participants reported the weather as both a limiting and a facilitating factor for PA in their weekly free-text assessments. Although the weather is not a modifiable element, persons with MS may benefit from advice on physical activities for rainy, snowy, and hot weather, as well as digital tools such as app-based personalized PA prescriptions for indoor exercises and activities [58].

Finally, our study also offers insights on a methodological level into best practices for sensor-based PA monitoring and PA barrier detection. Specifically, daily step count exhibited an inverse association with the BHADP score but only after adjustment for baseline step count levels. This finding is in line with previous literature, which has also described a relationship between a decrease in step count and an increase in the BHADP score [26]. By contrast, dichotomized analysis outcomes on the basis of the World Health Organization recommendation of 150 minutes of MVPA per week or the widely accepted threshold of 10,000 steps/d performed poorly in our analysis, likely in part owing to the loss of information through dichotomization. These observations suggest that intraindividual changes in PA may be more meaningful measures of PA barriers than absolute thresholds. Moreover, recent literature also suggests that PA <10,000 steps/d can improve health [10,59]. Therefore, we conducted a sensitivity analysis with a dichotomized threshold of 7000 steps/d, which did not materially alter our conclusions [10]. Accordingly, it may be more beneficial to monitor longitudinal within-person PA changes rather than goals set at fixed values.

### Limitations

Several limitations should be noted about this study. First, the sample size of the BarKA-MS study was restricted by recruitment potential and feasibility. Our analyses of the association between the BHADP score and the PA level may have been underpowered. The use of dichotomized outcomes in certain regressions further exacerbated the problem. In addition, through the aggregation of the Fitbit data at the daily level, PA fluctuations were missed [60]. PA at the daily level could reveal PA patterns, thus being more informative to better support persons with MS in PA engagement. Moreover, motivated by the explorative nature of the study, the analyses were not corrected for multiple testing. The BHADP scale was used to ascertain barriers to PA in persons with MS. However, the PA level is inevitably also influenced by the state of the disease. Therefore, it is highly likely that the items of the BHADP scale reflect both disease- and barrier-related differences simultaneously. In addition, we cannot exclude that personal interactions between persons with MS and staff at the rehabilitation clinic may have impacted perceived barriers also in the home setting (eg, through motivation or specific suggestions for home exercises). Furthermore, by assessing PA variation 4 weeks after a rehabilitation stay, our results are not representative of the long-term effect of a rehabilitation program on PA. Owing to the recruitment setting and the eligibility criteria applied, our results are not generalizable to the entire population of persons with MS in Switzerland. Finally, the presence of an on-site study coordinator during the completion of the baseline surveys and the surveys at the end of the rehabilitation stay may have led to information bias, especially in the well-being–related questionnaires (ie, barriers to PA, depression, walking ability, fatigue, health-related quality of life, pain, and self-efficacy).

### Conclusions

In summary, our data underscore the detrimental effect of common MS symptoms, including fatigue and depression, along with lifestyle and motivational barriers, on PA. Overcoming such barriers, particularly through more effective MS symptom management, may promote more active, healthier lifestyles. Furthermore, greater social support from family and friends could facilitate PA engagement in persons with MS. The involvement of close family members and friends in the care process might be a means to increase their support. Our study demonstrates that the BHADP scale is a valid and reliable instrument for assessing barriers to PA among persons with MS. Because of its association with the PA level of persons with MS, we encourage future use of the BHADP scale in combination with wearable fitness trackers to monitor and better support engagement in PA among persons with MS.

## Appendix

Multimedia Appendix 1 Methods complement detailing Fitbit data processing, free-text analysis methods, and missing data imputation. Results complement presenting sensitivity analyses based on either the complete case data set or a cut-off of 7,000 steps/d, providing insights into additional study time points and a different data granularity.

## Abbreviations

BarKA-MS: Barrieren für körperliche Aktivität bei Multiple Sklerosis-Betroffenen (Barriers to Physical Activity in People With Multiple Sclerosis)
BHADP: Barriers to Health Promoting Activities for Disabled Persons
EDSS: Expanded Disability Status Scale
FSMC: Fatigue Scale for Motor and Cognitive Functions
GSE: General Self-Efficacy Scale
MS: multiple sclerosis
MVPA: moderate to vigorous physical activity
PA: physical activity
PHQ-8: 8-item Patient Health Questionnaire depression scale
