# Supplementary material for: Exploring the Major Barriers to Physical Activity in Persons With Multiple Sclerosis: Observational Longitudinal Study
Source: JMIR Rehabil Assist Technol. 2024 Mar 18;11:e52733. doi: 10.2196/52733 (PMC10985607; doi:10.2196/52733)
Supplement: Multimedia Appendix 1 [file rehab_v11i1e52733_app1.docx]

**Multimedia Appendix of *Exploring the Major Barriers to Physical Activity in Persons With Multiple Sclerosis: Observational Longitudinal Study***

# Complement to the Methods

## Fitbit Data Processing

Missing Fitbit sensor data were detected and processed as follows. We defined non-wear time as 60 minutes or more of continuous inactivity: no step count, sedentary PA, and no heart rate information [30]. After excluding such non-wear time periods, daily measurements were considered valid if the Fitbit had been worn for at least 10 hours during waking hours between 6:00 a.m. and 11:00 p.m.

## Free-text Questions Analysis Methods

To verify whether the BHADP score includes the most relevant barriers and facilitators from a participant perspective, we performed a descriptive analysis of weekly participants’ feedback about barriers and facilitators to PA during the second phase of the study. The free-text entries were manually spell-checked and translated into English with DeepL Pro [31]. The package *udpipe*, version 0.8.9 [32,33] was used to part-of-speech tagging (i.e., their grammatical classification). The nouns and adjectives were retained and lemmatized. Word frequency bar plots were created using the R package *ggplot2*, version 3.3.6 (Figures S10 and S11 in Multimedia Appendix 1).

## Missing Data Imputation

Although study compliance and response rate to online surveys were very high in the BarKA-MS study [29], randomly missing data of <7% per data item were observed. The individual items were imputed by predictive mean matching on the basis of the other individual items within the same scale and the scale score [34]. The number of iterations was set at 50 based on the fraction of missing information [35]. Scale scores were manually recomputed after individual item imputation. Imputation was performed using the mice package, version 3.14.0 [36].

# Complement to the Results

**Table S1. Sensitivity analysis – Study participants characteristics.** Characteristics of the overall study sample (n=45) and comparison between the less active study participants (<7,000 steps/d; n=29) and the active study participants (≥7,000 steps/d; n=16).

| **Characteristics** | **Study participants**  **(n=45)** | **Study participants with <7,000 steps/d**  **(n=29)** | **Study participants with ≥7,000 steps/d**  **(n=16)** |
| --- | --- | --- | --- |
| **Baseline demographics** |  |  |  |
| **Sex** |  |  |  |
| Female | 29 (64.4%) | 17 (58.6%) | 12 (75%) |
| Male | 16 (35.6%) | 12 (41.4%) | 4 (25%) |
| **Age (y), median (IQR)** | 46 (40-51) | 50 (44-56) | 42 (40-46) |
| **Nationality^1^** |  |  |  |
| Swiss | 34 (75.6%) | 21 (72.4%) | 13 (81.2%) |
| German | 6 (13.3%) | 5 (17.2%) | 1 (6.2%) |
| Italian | 2 (4.4%) | 1 (3.4%) | 1 (6.2%) |
| Other | 3 (6.7%) | 2 (6.9%) | 1 (6.2%) |
| **Marital status** |  |  |  |
| Single | 12 (26.7%) | 8 (27.6%) | 4 (25%) |
| Married | 23 (51.1%) | 15 (51.7%) | 8 (50%) |
| Separated | 1 (2.2%) | 1 (3.4%) |  |
| Divorced | 7 (15.6%) | 4 (13.8%) | 3 (18.8%) |
| Widowed | 2 (4.4%) | 1 (3.4%) | 1 (6.2%) |
| **Education** |  |  |  |
| Mandatory school not completed (or up to and including grade 7) | 2 (4.4%) | 2 (6.9%) |  |
| Apprenticeship or Secondary education completed (i.e. Matura schools or intermediate diploma schools) | 25 (55.6%) | 16 (55.2%) | 9 (56.2%) |
| Higher professional education, Applied university or University completed | 18 (40%) | 11 (37.9%) | 7 (43.8%) |
| **Employment status** |  |  |  |
| Working full time | 5 (11.1%) | 4 (13.8%) | 1 (6.2%) |
| Working >50% but <100% | 5 (11.1%) | 4 (13.8%) | 1 (6.2%) |
| Working ≤50% or less | 17 (37.8%) | 12 (41.4%) | 5 (31.2%) |
| Not working | 18 (40%) | 9 (31%) | 9 (56.2%) |
| **Baseline health information** |  |  |  |
| **Multiple sclerosis type** |  |  |  |
| Relapsing-remitting multiple sclerosis | 18 (40%) | 8 (27.6%) | 10 (62.5%) |
| Primary-progressive multiple sclerosis | 8 (17.8%) | 5 (17.2%) | 3 (18.8%) |
| Secondary-progressive multiple sclerosis | 19 (42.2%) | 16 (55.2%) | 3 (18.8%) |
| **Multiple sclerosis duration (y), median (IQR)** | 11 (5-21) | 13 (5-23) | 11 (3-14) |
| **Expanded Disability Status Scale score, median (IQR)** | 4.5 (3.5-6) | 5 (4-6) | 3.5 (3-4) |
| **Expanded Disability Status Scale score** |  |  |  |
| 0-3.5 | 15 (33.3%) | 6 (20.7%) | 9 (56.2%) |
| 4-5.5 | 18 (40%) | 13 (44.8%) | 5 (31.2%) |
| ≥6 | 12 (26.7%) | 10 (34.5%) | 2 (12.5%) |
| **Time since last relapse (y), median (IQR)** | 3 (1-5) | 3 (1-12) | 2 (1.5-4) |
| Missing information, n (%) | 8 (17.8%) | 7 (15.6%) | 1 (2.2%) |
| **BMI (kg/m^2^), median (IQR)** | 24 (21-28) | 24 (22-26) | 23.5 (20.8-29.2) |
| Missing information, n(%) | 0 (0%) | 0 (0%) | 0 (0%) |
| **BMI (kg/m^2^), n(%)** |  |  |  |
| <18.5 (underweight ) | 5 (11.1%) | 3 (10.3%) | 2 (12.5%) |
| 18.5-24.9 (healthy weight ) | 22 (48.9%) | 16 (55.2%) | 6 (37.5%) |
| 25.0-29.9 (overweight ) | 10 (22.2%) | 6 (20.7%) | 4 (25%) |
| ≥30.0 (obesity ) | 8 (17.8%) | 4 (13.8%) | 4 (25%) |
| **Comorbidities^1^** |  |  |  |
| None | 18 (40%) | 12 (41.4%) | 6 (37.5%) |
| Hypertension | 5 (11.1%) | 5 (17.2%) | 0 (0%) |
| Depression | 5 (11.1%) | 4 (13.8%) | 1 (6.2%) |
| Skin diseases (e.g., acne) | 4 (8.9%) | 1 (3.4%) | 3 (18.8%) |
| Orthopedic diseases (e.g., joint or back pain) | 4 (8.9%) | 3 (10.3%) | 1 (6.2%) |
| Type 2 diabetes | 3 (6.7%) | 2 (6.9%) | 1 (6.2%) |
| Migraine | 2 (4.4%) |  | 2 (12.5%) |
| Hypothyroidism | 2 (4.4%) |  | 2 (12.5%) |
| Other^2^ | 9 (20%) | 7 (24.1%) | 2 (12.5%) |
| **Change in the amount of sport practiced after the multiple sclerosis diagnosis, n (%)** | | | |
| Less | 27 (60%) | 20 (69%) | 7 (43.8%) |
| Same amount | 2 (4.4%) | 1 (3.4%) | 1 (6.2%) |
| More | 15 (33.3%) | 7 (24.1%) | 8 (50%) |
| Missing information | 1 (2.2%) | 1 (3.4%) |  |
| **Time spent at the rehabilitation clinic (d), median (IQR)** | 22 (18-26) | 22 (18-26) | 22 (19-24) |
| **Barriers to Health Promoting Activities for Disabled Persons scale score *at analysis baseline*** (ie, at the end of rehabilitation stay; range 18-72, the higher the score, the more barriers to physical activity), median (IQR) | 20 (19-21) | 20 (19-22) | 20 (19-21) |
| **End-of-study assessments** |  |  |  |
| **Barriers to Health Promoting Activities for Disabled Persons scale score *at the end of the study*** (score range 18-72, the higher the score, the more barriers to physical activity), median (IQR) | 28 (24-35) | 29 (22-35) | 28 (25-33) |
| **12-item Multiple Sclerosis Walking Scale score** (range 0-100, the higher the score, the lower the walking ability), median (IQR) | 45.8 (29.2-79.2) | 64.6 (43.8-85.4) | 29.2 (27.1-34.4) |
| Missing information, n (%) | 6 (13.3%) | 4 (8.9%) | 2 (4.4%) |
| **Fatigue Scale for Motor and Cognitive Functions score** (range 20-100, the higher the score, the more the fatigue), n (%) |  |  |  |
| <43 (no fatigue) | 9 (20%) | 7 (24.1%) | 2 (12.5%) |
| 43-52 (mild fatigue) | 6 (13.3%) | 4 (13.8%) | 2 (12.5%) |
| 53-62 (moderate fatigue) | 8 (17.8%) | 3 (10.3%) | 5 (31.2%) |
| ≥63 (severe fatigue) | 15 (33.3%) | 11 (37.9%) | 4 (25%) |
| Missing information | 7 (15.6%) | 4 (13.8%) | 3 (18.8%) |
| **Fatigue Scale for Motor and Cognitive Functions - Cognitive fatigue score** (range 10-50, the higher the score, the more the fatigue), n (%) |  |  |  |
| <22 (no cognitive fatigue) | 17 (37.8%) | 12 (41.4%) | 5 (31.2%) |
| 22-27 (mild cognitive fatigue) | 6 (13.3%) | 3 (10.3%) | 3 (18.8%) |
| 28-33 (moderate cognitive fatigue) | 8 (17.8%) | 4 (13.8%) | 4 (25%) |
| ≥34 (severe cognitive fatigue) | 9 (20%) | 7 (24.1%) | 2 (12.5%) |
| Missing information | 5 (11.1%) | 3 (10.3%) | 2 (12.5%) |
| **Fatigue Scale for Motor and Cognitive Functions - Motor fatigue** **score** (range 10-50, the higher the score, the more the fatigue) , n (%) |  |  |  |
| <22 (no motor fatigue) | 6 (13.3%) | 5 (17.2%) | 1 (6.2%) |
| 22-26 (mild motor fatigue) | 4 (8.9%) | 2 (6.9%) | 2 (12.5%) |
| 27-31 (moderate motor fatigue) | 9 (20%) | 4 (13.8%) | 5 (31.2%) |
| ≥32 (severe motor fatigue) | 22 (48.9%) | 16 (55.2%) | 6 (37.5%) |
| Missing information | 4 (8.9%) | 2 (6.9%) | 2 (12.5%) |
| **General Self-Efficacy Scale score** (range 10-40, the higher the score, the more the self-efficacy), median (IQR) | 32 (30-36) | 32 (29-36) | 31 (30-35) |
| **8-item Patient Health Questionnaire Depression Scale score** (range 0-24, the higher the score, the more the depression signs), n (%) |  |  |  |
| <10 (not clinically significant depression) | 35 (77.8%) | 20 (69%) | 15 (93.8%) |
| ≥ 10 (clinically significant depression) | 7 (15.6%) | 7 (24.1%) |  |
| Missing information | 3 (6.7%) | 2 (6.9%) | 1 (6.2%) |
| **EuroQol 5-Dimension 5-Level weighted by the French values set** ( range 0-100, the higher the score, the better the quality of life), median (IQR) | 63.5 (45.6-78.8) | 62.9 (39.8-72.3) | 74.7 (63.2-85.8) |
| Missing information | 2 (4.4%) | 1 (2.2%) | 1 (2.2%) |
| **“How bad was your pain when it was at its worst during the last 7 days?”** (visual analog scale; range 0-10, the higher the score, the greater the pain), median (IQR) | 3 (0-6) | 3 (0-7) | 3 (0-4) |
| ^1^Multiple answers possible  ^2^Asthma, type 1 diabetes, osteoporosis, psoriasis, cancer, rheumatic diseases, elevated cholesterol level, colitis ulcerosa, Fibromyalgia, Shingles, Meniere disease, and cerebellar syndrome |  |  |  |


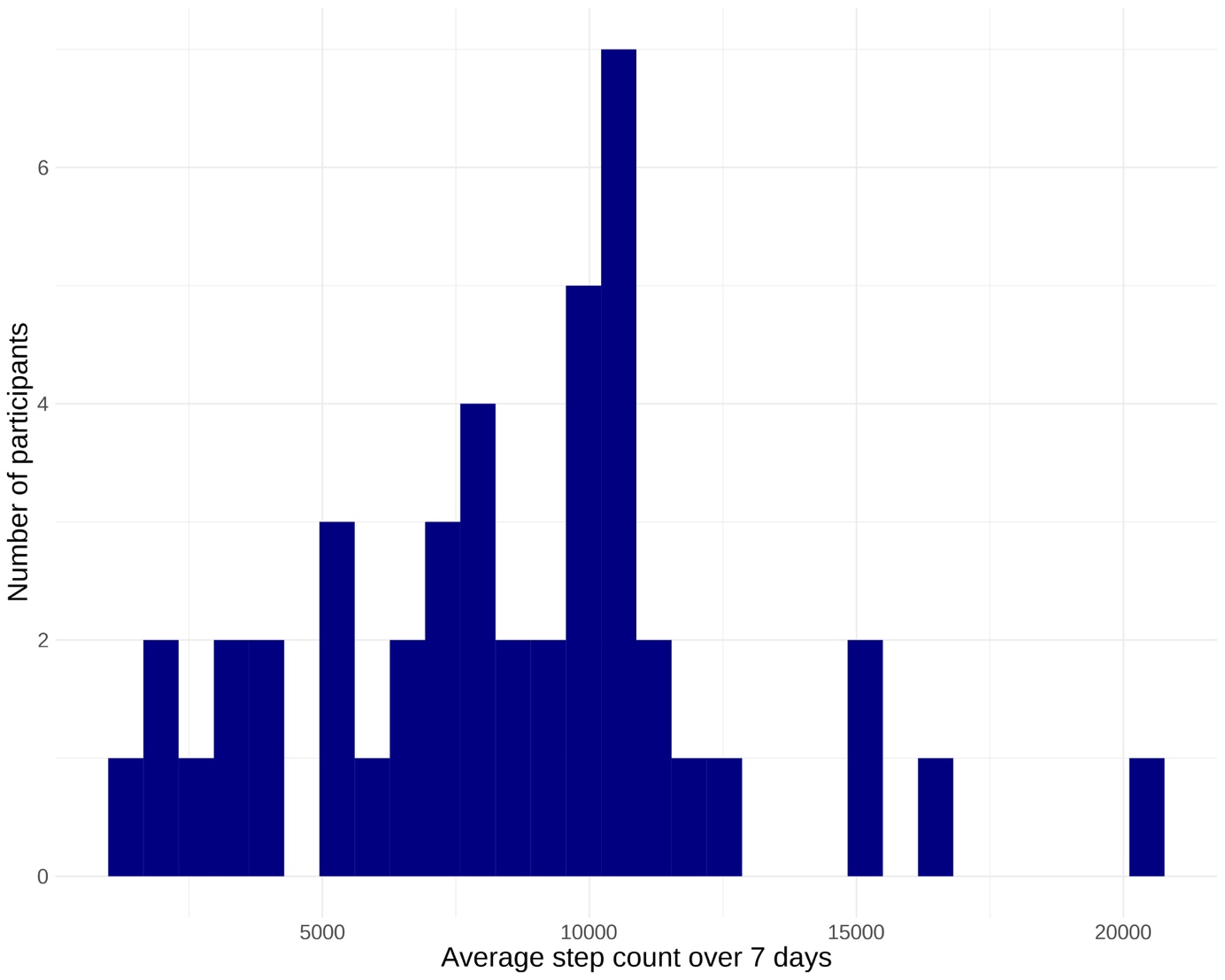
**Figure S1. 7-day steps distribution in rehabilitation.** Distribution of the average number of steps walked by the study participants (n=45) during the last week of rehabilitation.

**
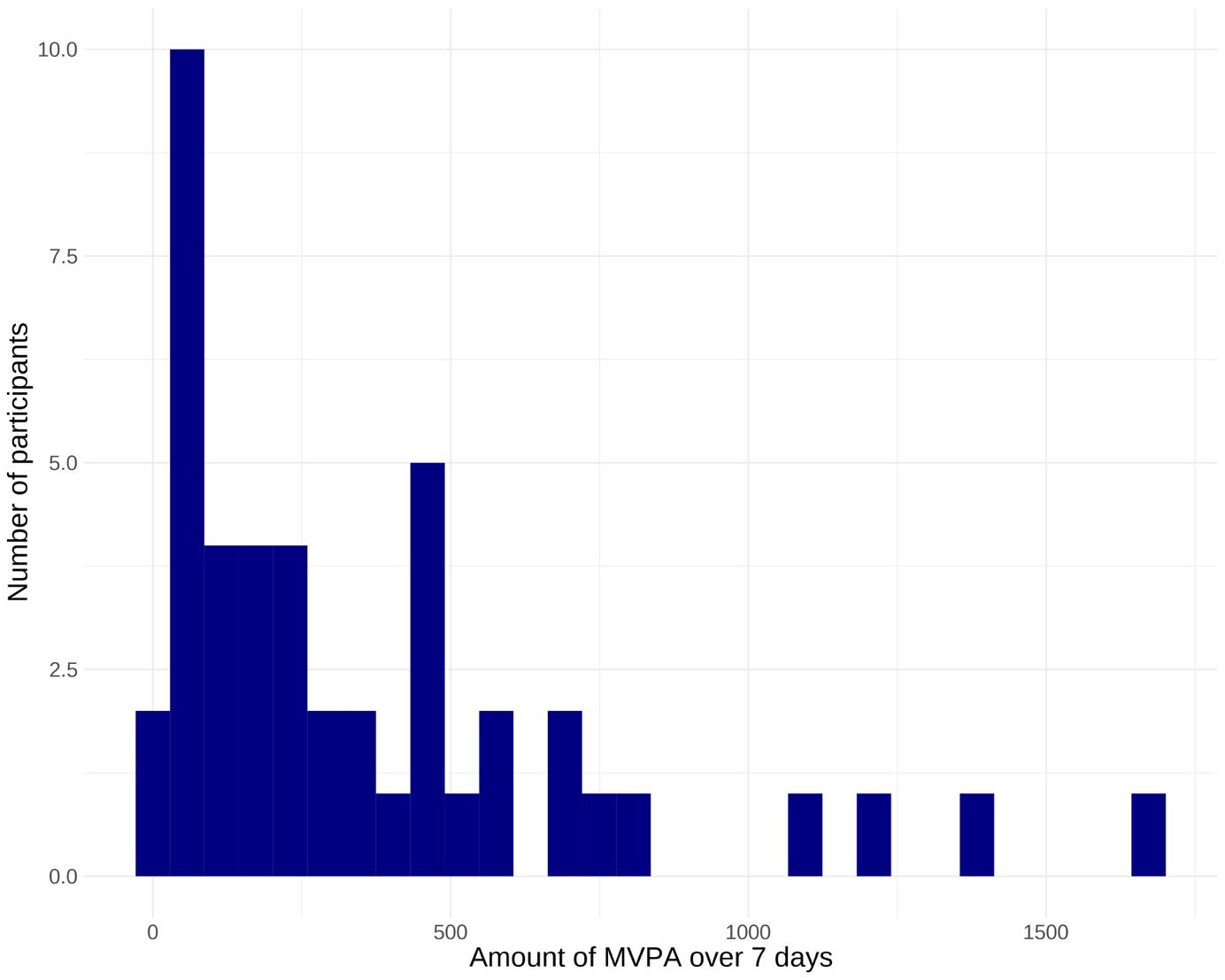
Figure S2. 7-day MVPA distribution in rehabilitation.** Distribution of the average number of minutes of MVPA achieved by the study participants (n=45) during the last week of rehabilitation.


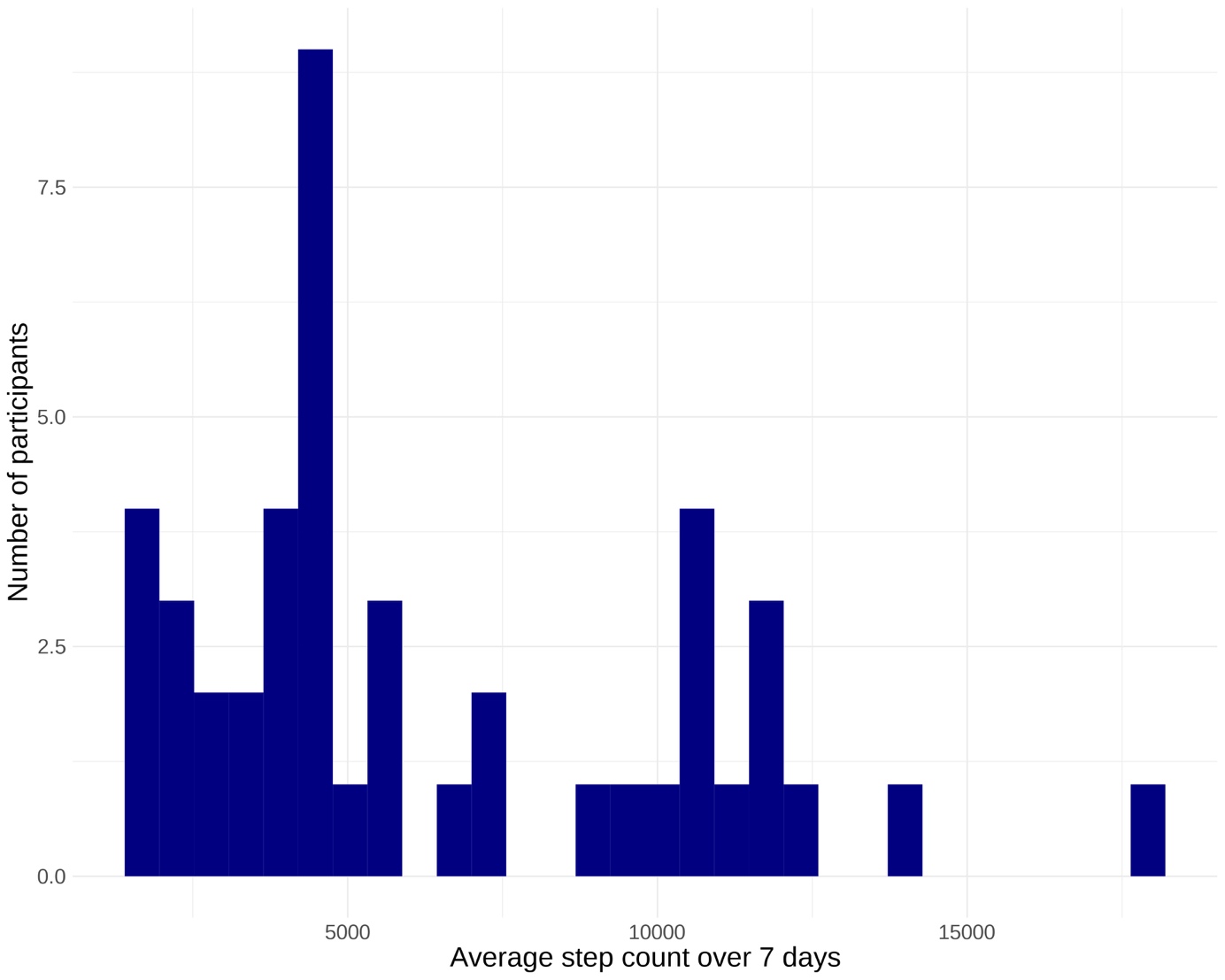
**Figure S3. 7-day steps distribution at end-of-study.** Distribution of the average number of steps walked by the study participants (n=45) during the last week of the study.

**
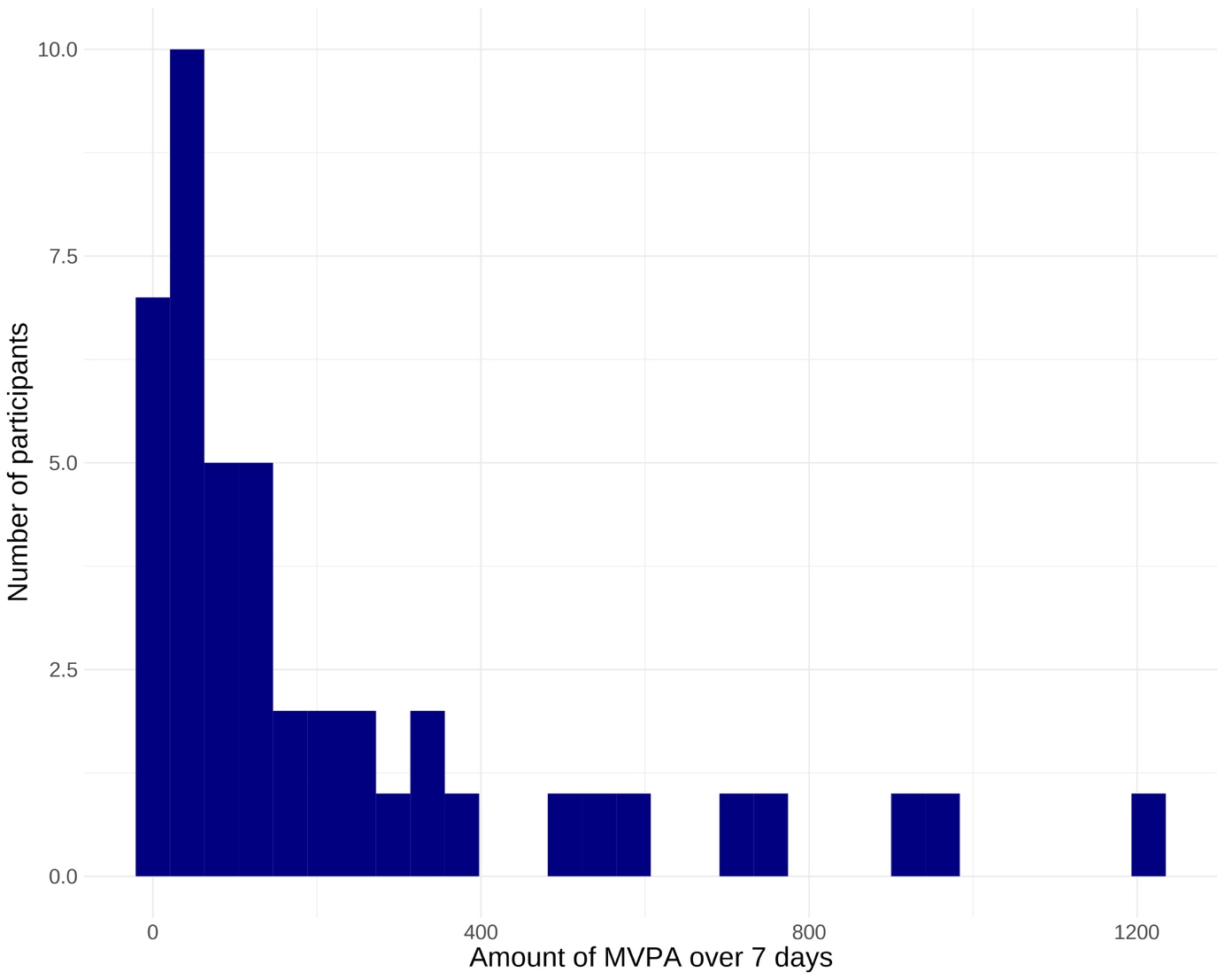
Figure S4. 7-day MVPA distribution at end-of-study.** Distribution of the average number of minutes of MVPA achieved by the study participants (n=45) during the last week of the study.

**Table S2. Barriers to physical activity by physical activity level.** Mean, standard deviation, t-statistic, and p-value of the average score of the 18 items of the Barriers to Health Promoting Activities for Disabled Persons Scale (item score range: 1-4) reported at the end of the study by the less active participants (<10,000 steps/d; n=33) and the active participants (≥10,000 steps/d; n=12), in decreasing order for the participants with <10,000 steps/d and displayed in Figure 1. Higher scores reflect greater barriers; p-values ≤0.05 are in bold and marked by a *.

|  |  | **<10,000 steps/d** | | **≥10,000 steps/d** | | **t-statistic** | **p-value** |
| --- | --- | --- | --- | --- | --- | --- | --- |
| **Item #** | **Barriers** | **Mean** | **Standard deviations** | **Mean** | **Standard deviations** |  |  |
| **6** | **Impairment** | 2.5 | 1 | 2 | 0.7 | 1.8 | 0.09 |
| **2** | **Too tired** | 2.4 | 0.9 | 2.2 | 0.9 | 0.6 | 0.56 |
| **13** | **Interferes with other responsibilities** | 1.9 | 0.9 | 2.1 | 0.9 | -0.5 | 0.64 |
| **17** | **Bad weather** | 1.9 | 0.6 | 1.6 | 0.5 | 1.6 | 0.12 |
| **1** | **Lack of convenient facilities** | 1.8 | 0.7 | 2 | 0.9 | -0.7 | 0.51 |
| **14** | **Lack of time** | 1.7 | 0.9 | 2.1 | 0.9 | -1.2 | 0.25 |
| **4** | **Feeling what I do doesn't help** | 1.6 | 0.7 | 1.2 | 0.4 | 2.6 | **0.01*** |
| **5** | **Lack of money** | 1.6 | 0.8 | 1.6 | 0.7 | 0 | 0.98 |
| **11** | **Concern about safety** | 1.6 | 0.7 | 1.5 | 0.7 | 0.1 | 0.90 |
| **3** | **Lack of transportation** | 1.5 | 0.7 | 1.6 | 1 | -0.4 | 0.72 |
| **7** | **No one to help me** | 1.5 | 0.6 | 1.1 | 0.3 | 3 | **0.005*** |
| **8** | **Not interested** | 1.5 | 0.5 | 1.3 | 0.5 | 0.7 | 0.48 |
| **15** | **Feeling I can't do things correctly** | 1.5 | 0.6 | 1.2 | 0.6 | 1.5 | 0.14 |
| **12** | **Lack of support from family/friends** | 1.4 | 0.7 | 1 | 0 | 3.2 | **0.003*** |
| **9** | **Lack of information** | 1.3 | 0.5 | 1.2 | 0.4 | 0.7 | 0.47 |
| **10** | **Embarrassment about my appearance** | 1.3 | 0.6 | 1.2 | 0.4 | 0.9 | 0.38 |
| **16** | **Difficulty with communication** | 1.3 | 0.6 | 1.2 | 0.4 | 0.6 | 0.57 |
| **18** | **Lack of help from healthcare professionals** | 1.1 | 0.2 | 1.1 | 0.3 | -0.2 | 0.81 |

The computations were conducted on the complete case dataset. There were missing values in the following scale items: *lack of convenient facilities* (1), *too tired* (2), *lack of transportation* (1), *no one to help me* (1), *concern about safety* (1), *feeling I can’t do things correctly* (2), and *difficulty with communication* (2).


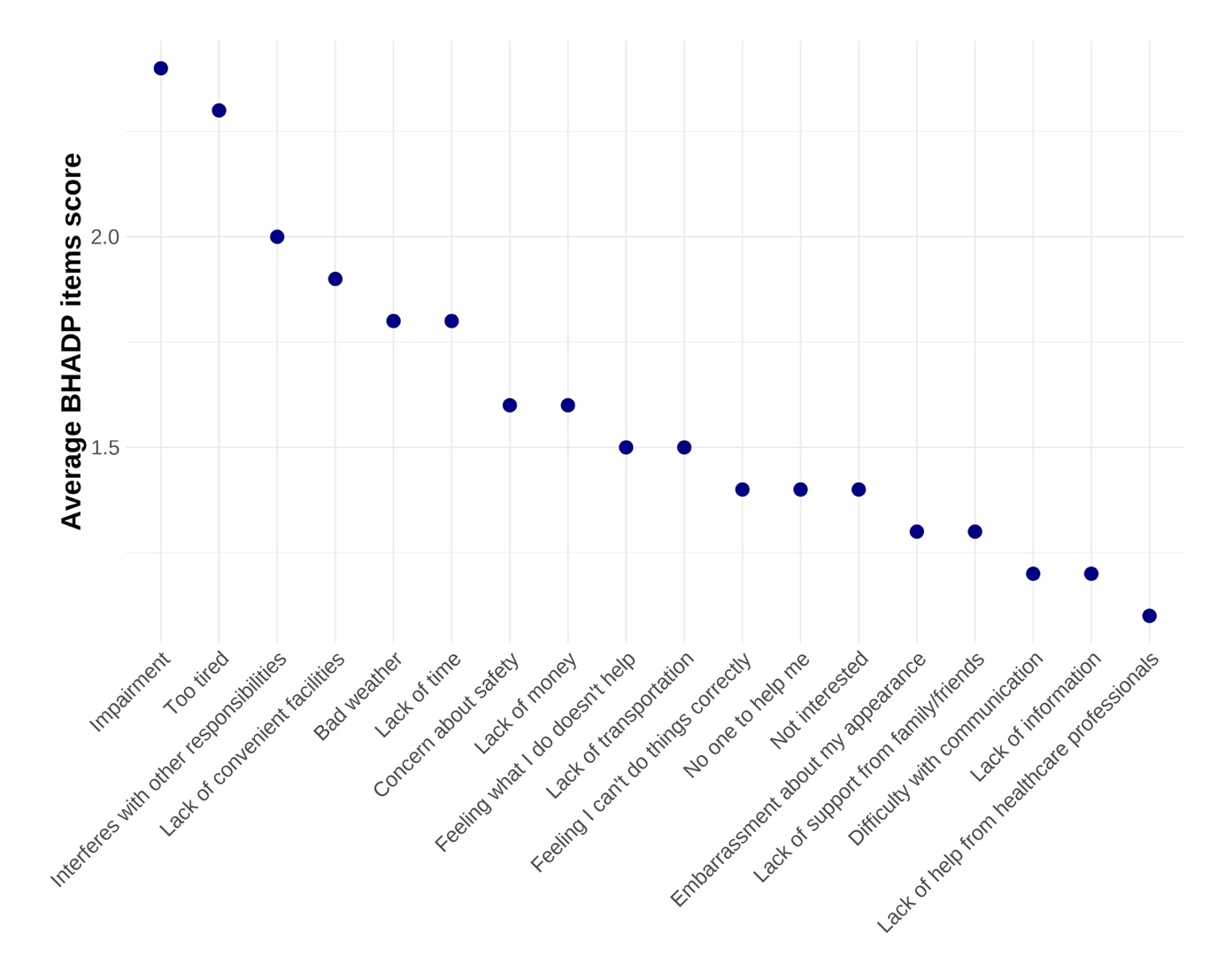
**Figure S5. Barriers to physical activity.** Average score of the 18 items of the Barriers to Health Promoting Activities for Disabled Persons Scale (item score range: 1-4) reported at the end of the study by the study participants (n=45), in decreasing order.

The figure is based on the complete case dataset. There were missing values in the following scale items: *lack of convenient facilities* (1), *too tired* (2), *lack of transportation* (1), *no one to help me* (1), *concern about safety* (1), *feeling I can’t do things correctly* (2), and *difficulty with communication* (2).

BHADP: Barriers to Health Promoting Activities for Disabled Persons

**Table S3. Barriers to physical activity.** Mean and standard deviation of the average score of the 18 items of the Barriers to Health Promoting Activities for Disabled Persons Scale (item score range: 1-4) reported at the end of the study by the study participants (n=45), in decreasing order and displayed in Figure 2. Higher scores reflect greater barriers.

| **Item #** | **Barriers** | **Mean** | **Standard deviation** |
| --- | --- | --- | --- |
| **6** | **Impairment** | 2.4 | 0.9 |
| **2** | **Too tired** | 2.3 | 0.9 |
| **13** | **Interferes with other responsibilities** | 2 | 0.9 |
| **1** | **Lack of convenient facilities** | 1.9 | 0.7 |
| **14** | **Lack of time** | 1.8 | 0.9 |
| **17** | **Bad weather** | 1.8 | 0.6 |
| **5** | **Lack of money** | 1.6 | 0.8 |
| **11** | **Concern about safety** | 1.6 | 0.7 |
| **3** | **Lack of transportation** | 1.5 | 0.8 |
| **4** | **Feeling what I do doesn't help** | 1.5 | 0.7 |
| **7** | **No one to help me** | 1.4 | 0.6 |
| **8** | **Not interested** | 1.4 | 0.5 |
| **15** | **Feeling I can't do things correctly** | 1.4 | 0.6 |
| **10** | **Embarrassment about my appearance** | 1.3 | 0.5 |
| **12** | **Lack of support from family/friends** | 1.3 | 0.6 |
| **9** | **Lack of information** | 1.2 | 0.5 |
| **16** | **Difficulty with communication** | 1.2 | 0.6 |
| **18** | **Lack of help from healthcare professionals** | 1.1 | 0.3 |

The computations were conducted on the complete case dataset. There were missing values in the following scale items: *lack of convenient facilities* (1), *too tired* (2), *lack of transportation* (1), *no one to help me* (1), *concern about safety* (1), *feeling I can’t do things correctly* (2), and *difficulty with communication* (2).

**
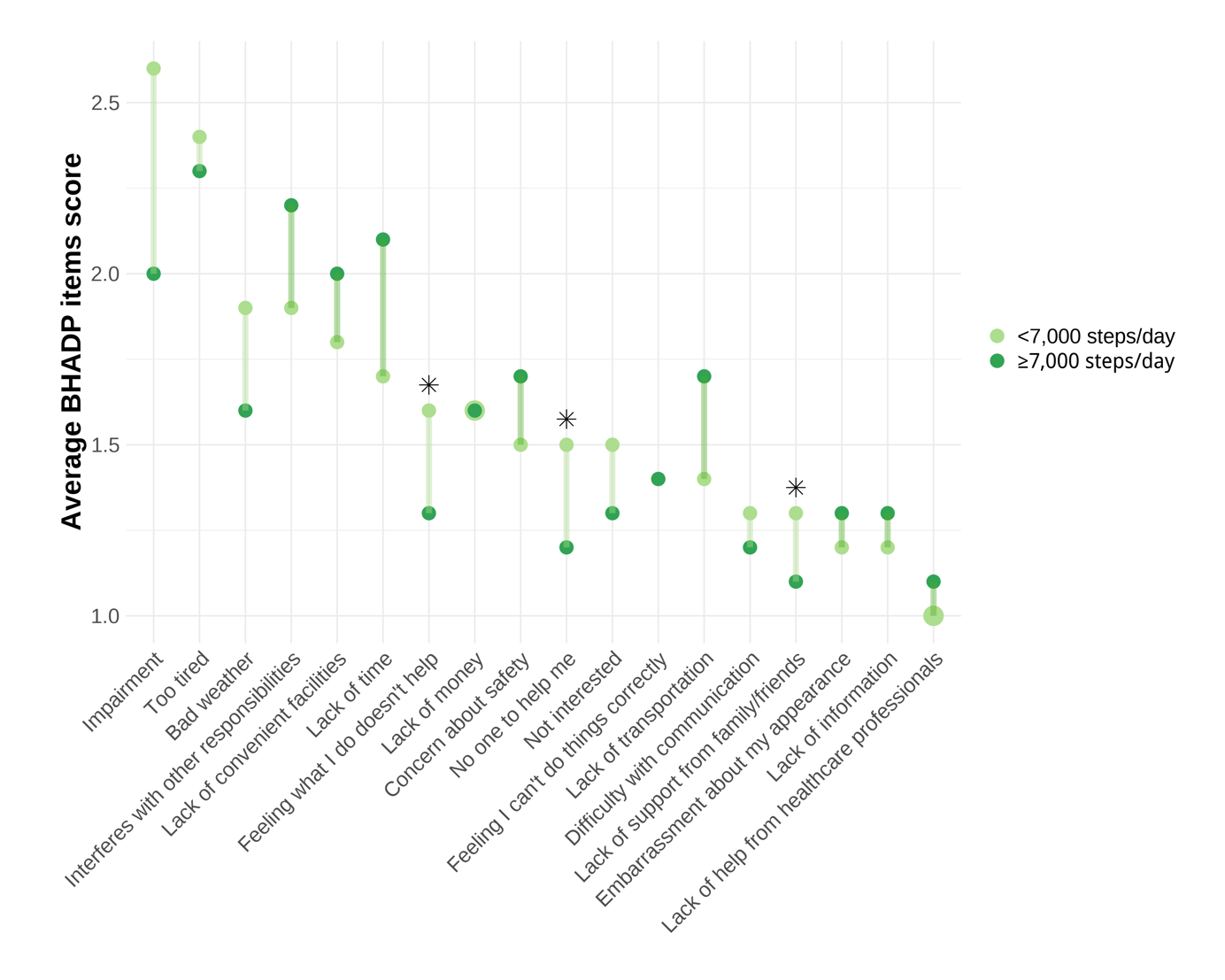
Figure S6 Barriers to physical activity by physical activity level.** Average score of the 18 items of the Barriers to Health Promoting Activities for Disabled Persons Scale (item score range: 1-4) reported at the end of the study by the less active participants (<7,000 steps/day; n=29; in light green) and the active participants (≥7,000 steps/day; n=16; in dark green), in decreasing order for the less active participants. The difference between both groups is colored with the color corresponding to the group with the highest score. Statistically significant differences (*P*-value <0.05) are marked by a “*”. Higher scores reflect greater barriers.


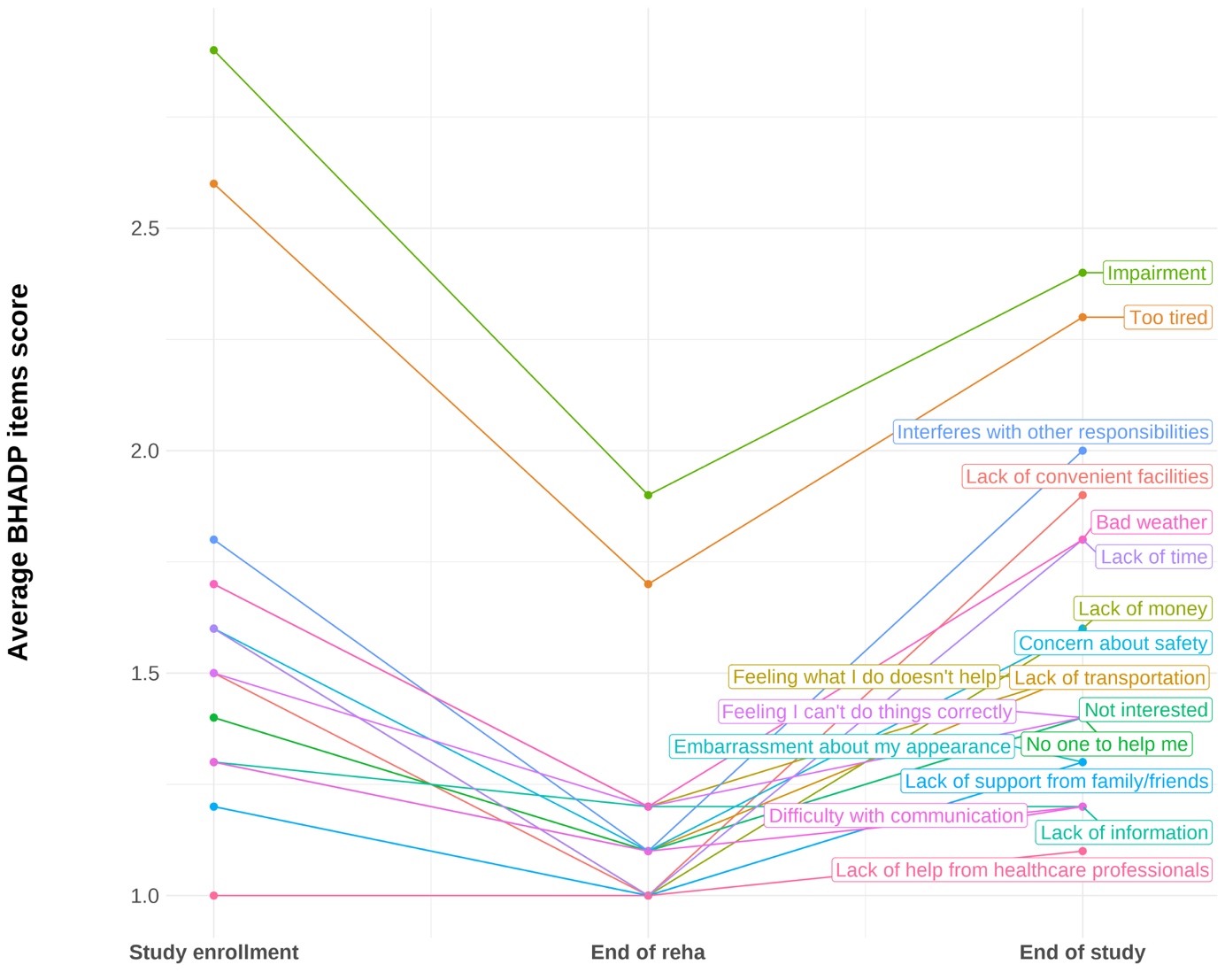
**Figure S7. Barriers to physical activity over time.** Average score of the 18 items of the Barriers to Health Promoting Activities for Disabled Persons Scale (item score range: 1-4) reported at study enrollment, the end of rehabilitation stay, and the end of the study by the study participants (n=45). Higher scores reflect greater barriers.

The computations were conducted on the complete case dataset. There were missing values in the following scale items:

Study enrollment: no missing values;

End of rehabilitation: *feeling what I do doesn’t help* (1), *lack of support from family/friends* (1);

End of study: *lack of convenient facilities* (1), *too tired* (2), *lack of transportation* (1), 1 *no one to help me* (1), *concern about safety* (1), *feeling I can’t do things correctly* (2), and *difficulty with communication* (2).


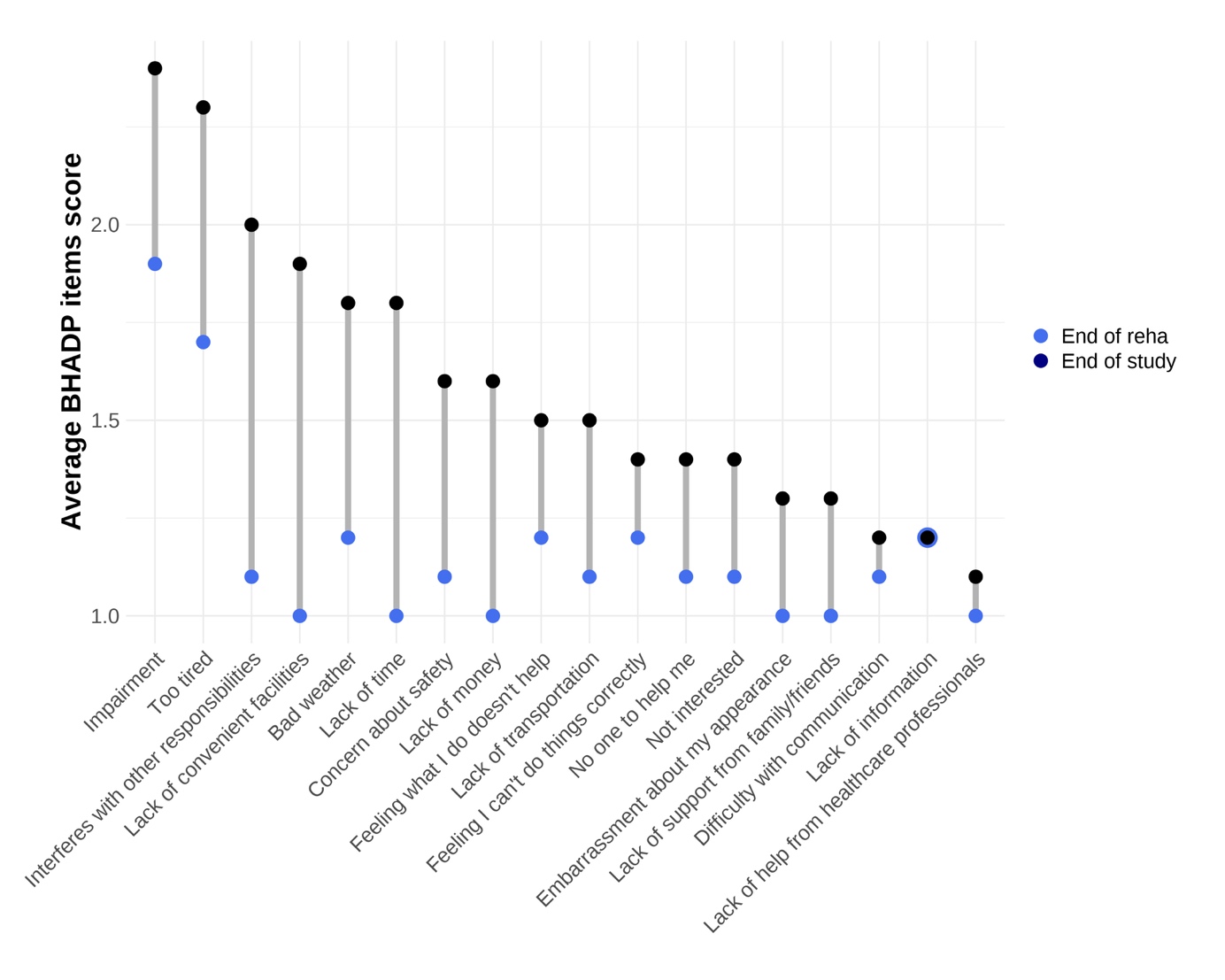
**Figure S8. Barriers to physical activity: end of rehabilitation vs end of study.** Average score of the 18 items of the Barriers to Health Promoting Activities for Disabled Persons Scale (item score range: 1-4) reported at the end of the rehabilitation stay (medium blue) and at the end of the study (dark blue) by the study participants (n=45), in decreasing order for the scores reported at the end of the study. The difference between both groups is colored with the color corresponding to the group with the highest score. Higher scores reflect greater barriers.

The computations were conducted on the complete case dataset. There were missing values in the following scale items:

End of rehabilitation: *feeling what I do doesn’t help* (1), *lack of support from family/friends* (1);

End of study: *lack of convenient facilities* (1), *too tired* (2), *lack of transportation* (1), 1 *no one to help me* (1), *concern about safety* (1), *feeling I can’t do things correctly* (2), and *difficulty with communication* (2).


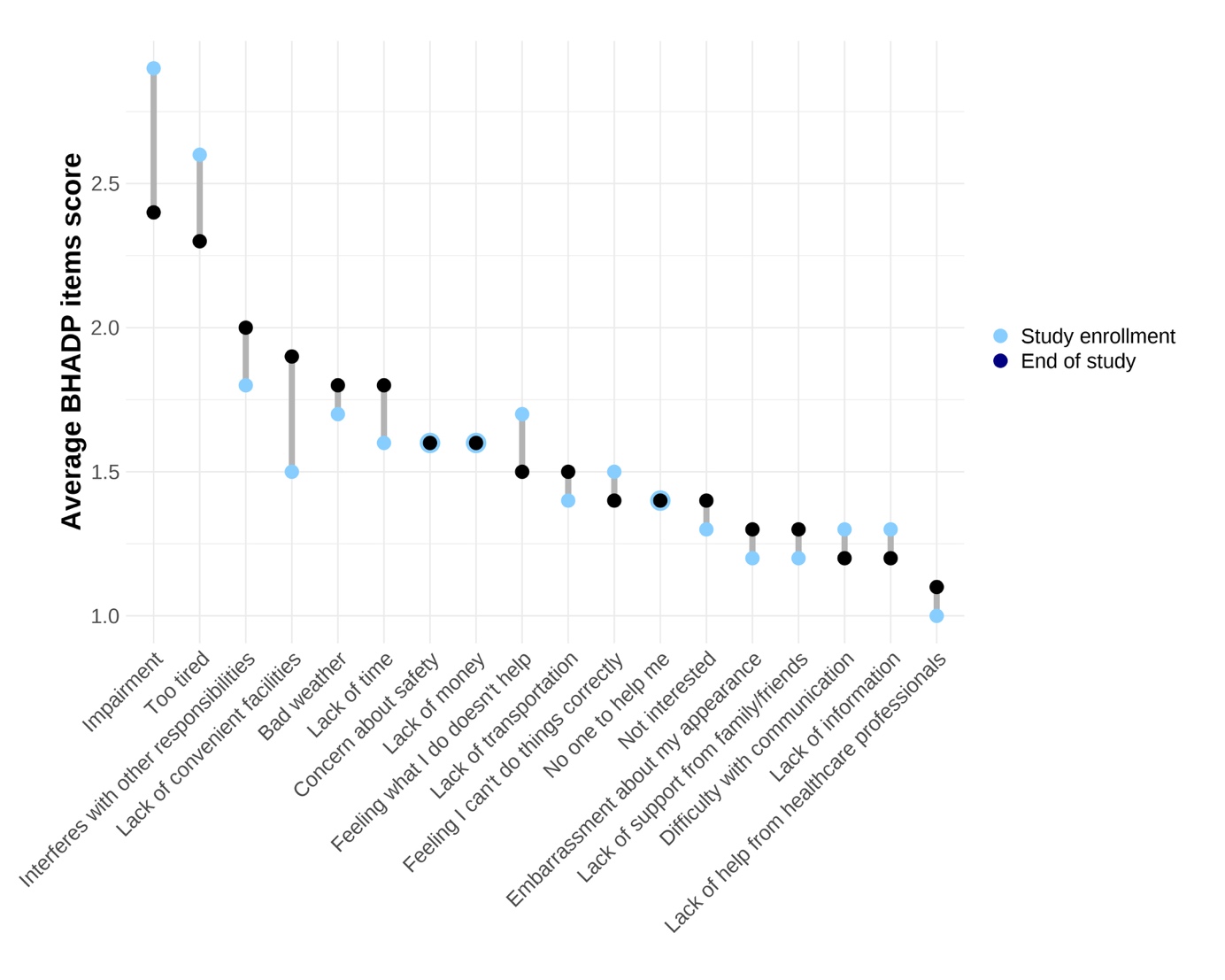
**Figure S9. Barriers to physical activity: study enrollment vs end of study.** Average score of the 18 items of the Barriers to Health Promoting Activities for Disabled Persons Scale (item score range: 1-4) reported at study enrollment (blue sky) and at the end of the study (dark blue) by the study participants (n=45), in decreasing order for the scores reported at the end of the study. The difference between both groups is colored with the color corresponding to the group with the highest score. Higher scores reflect greater barriers.

The computations were conducted on the complete case dataset. There were missing values in the following scale items:

Study enrollment: no missing values;

End of study: *lack of convenient facilities* (1), *too tired* (2), *lack of transportation* (1), 1 *no one to help me* (1), *concern about safety* (1), *feeling I can’t do things correctly* (2), and *difficulty with communication* (2).


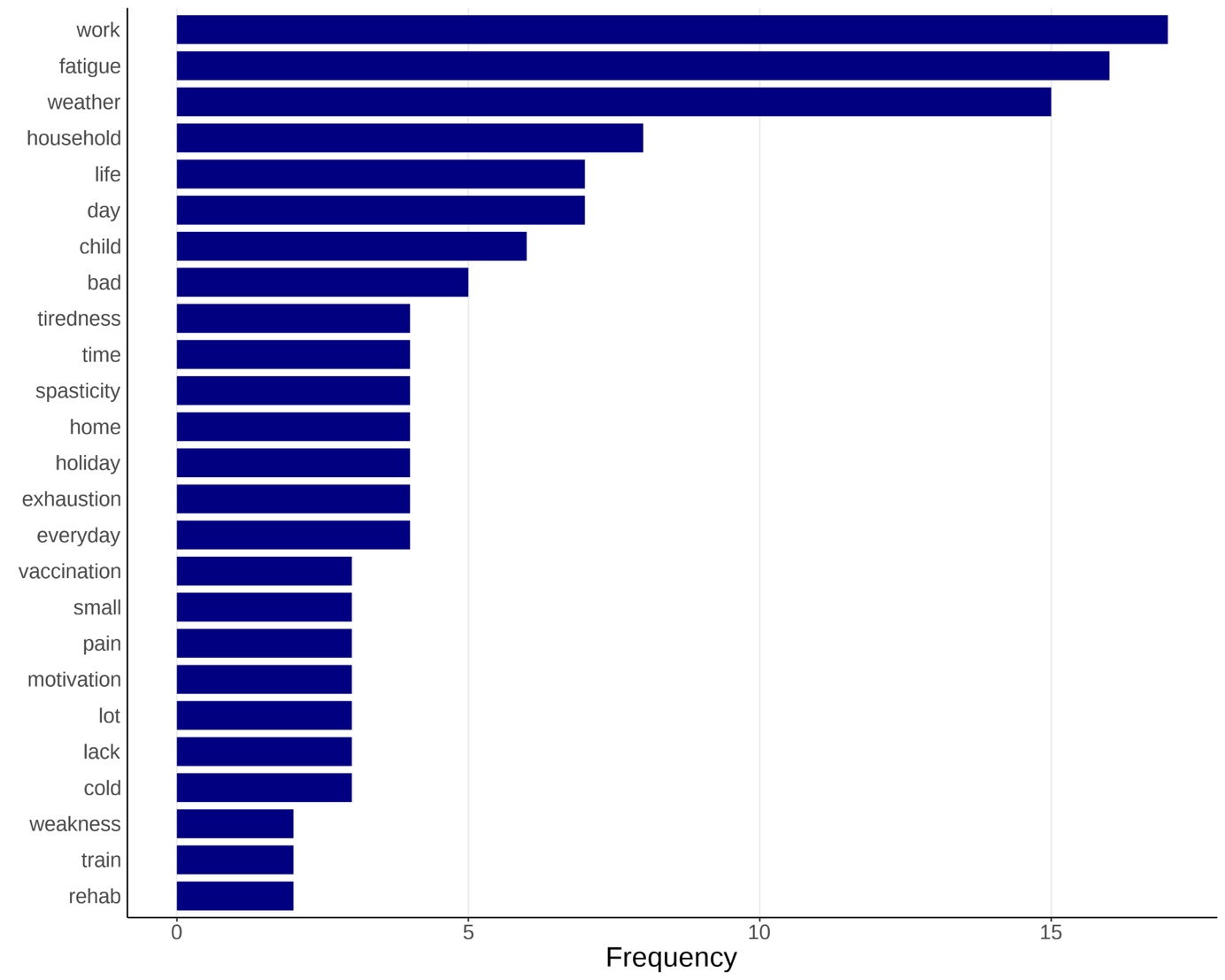
**Figure S10. Barriers to physical activity.** Word frequency of the 25 most frequent words used in the answers to the weekly question “What kept you from being physically active this week?” during the home phase (n answers = 95).

**
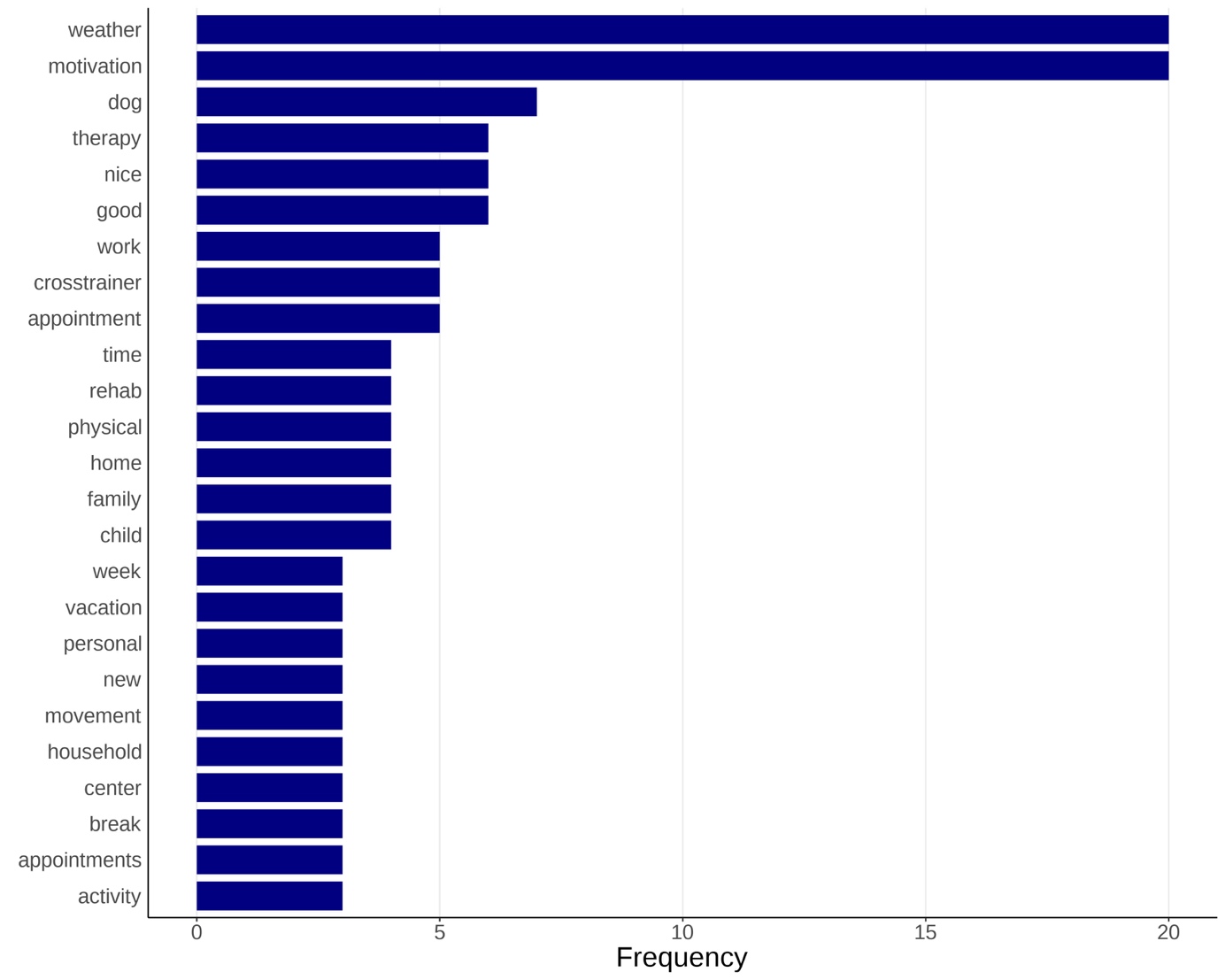
Figure S11. Facilitators to physical activity.** Word frequency of the 25 most frequent words used in the answers to the weekly question “What made it easier for you to be physically active this week?” during the home phase (n answers = 128).


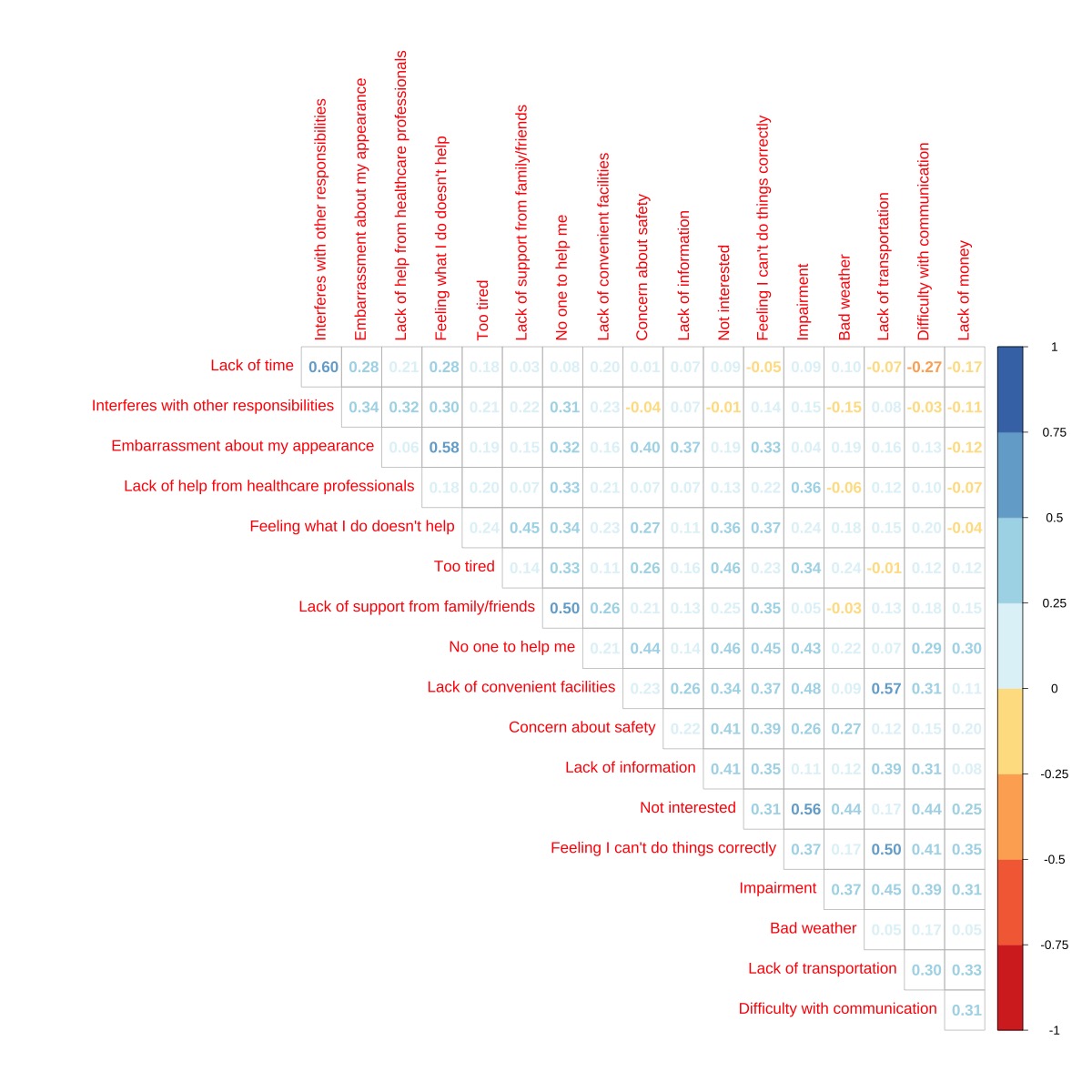
**Figure S12. BHADP items correlation.** Correlation of the 18 items of the Barriers to Health Promoting Activities for Disabled Persons Scale score among themselves.

The computations were conducted on the complete case dataset.

**Figure S13. BHADP score correlation with well-being scores.** Correlation of the Barriers to Health Promoting Activities for Disabled Persons Scale score with various well-being **
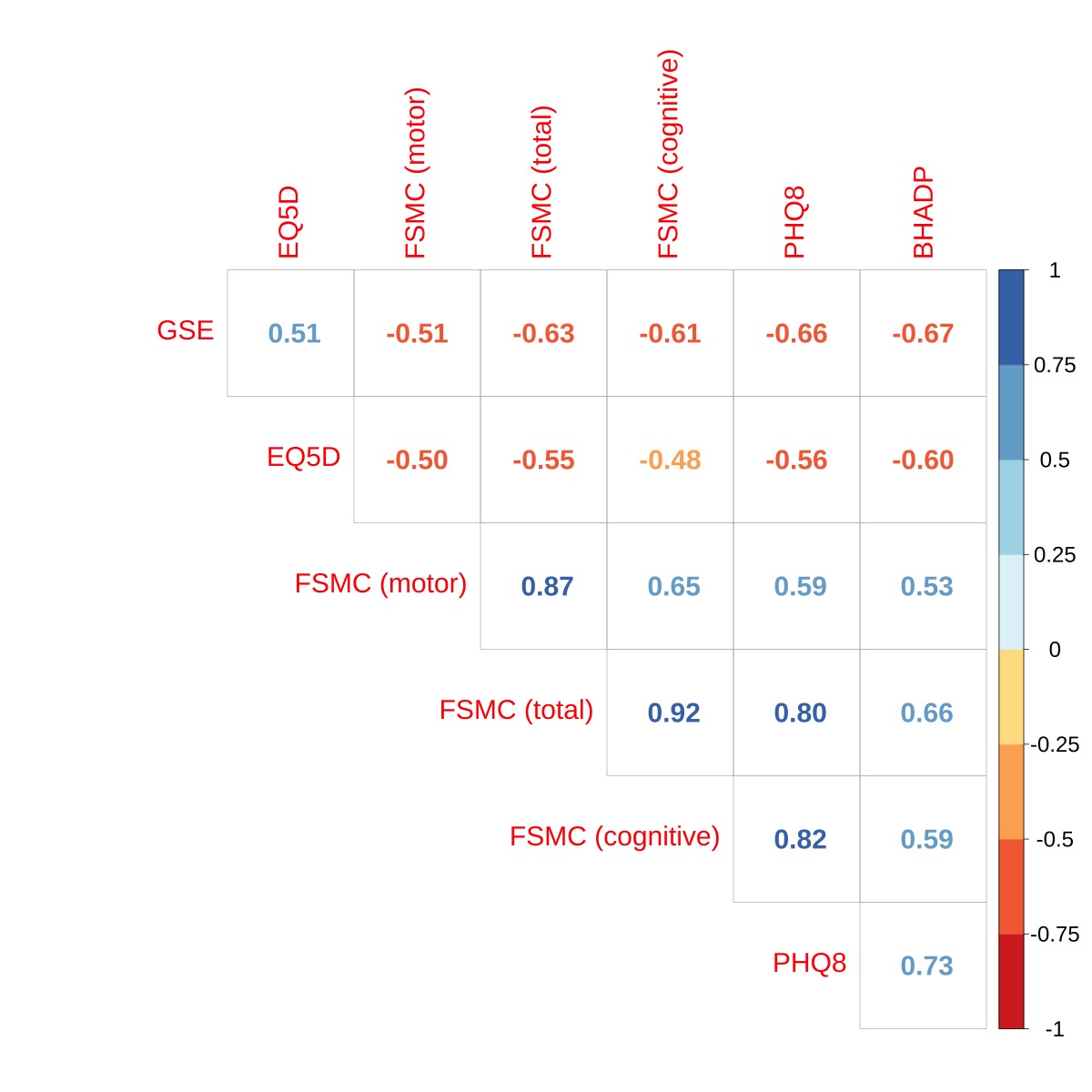
**questionnaires.

The computations were conducted on the complete case dataset.

BHADP: Barriers to Health Promoting Activities for Disabled Persons scale; EQ5D: EuroQol-5 Dimension; FSMC: Fatigue Scale for Motor and Cognitive Functions scale; GSE: General Self-Efficacy scale; PHQ8: eight-item Patient Health Questionnaire depression scale.

**Table S4. Sensitivity analysis – Linear regression analyses with the BHADP score as outcome.** Confounder adjusted linear regression models to assess the association of the BHADP scale score (dependent variable) with the PHQ8, FSMC, EQ5D, and GSE scale scores (independent variables), respectively, based on the complete cases dataset (n=29).

|  | **BHADP**^1^ **score vs. PHQ8**^2^ | | | **BHADP**^1^ **score vs. FSMC**^3^ | | | **BHADP**^1^ **score vs. EQ5D**^4^ | | | **BHADP**^1^ **score vs. GSE**^5^ | | |
| --- | --- | --- | --- | --- | --- | --- | --- | --- | --- | --- | --- | --- |
| **Characteristic** | **Beta** | **95% CI^1^** | **p-value** | **Beta** | **95% CI^1^** | **p-value** | **Beta** | **95% CI^1^** | **p-value** | **Beta** | **95% CI^1^** | **p-value** |
| **(Intercept)** | 29 | 19, 39 | <0.001 | 22 | 10, 35 | <0.001 | 56 | 43, 70 | <0.001 | 52 | 39, 66 | <0.001 |
| **Age** | -0.03 | -0.22, 0.15 | 0.7 | -0.01 | -0.21, 0.18 | 0.9 | 0.02 | -0.20, 0.23 | 0.9 | 0.04 | -0.20, 0.27 | 0.7 |
| **Sex** |  |  |  |  |  |  |  |  |  |  |  |  |
| Female | — | — |  | — | — |  | — | — |  | — | — |  |
| Male | 2.4 | -1.2, 6.0 | 0.2 | 2.7 | -1.2, 6.5 | 0.2 | 3.8 | -0.32, 7.8 | 0.069 | 4.5 | 0.12, 8.8 | 0.044 |
| **BMI**^7^ | -0.14 | -0.40, 0.13 | 0.3 | -0.25 | -0.52, 0.02 | 0.066 | -0.45 | -0.75, -0.15 | 0.005 | -0.28 | -0.60, 0.04 | 0.083 |
| **MS^8^ duration** | -0.05 | -0.23, 0.12 | 0.5 | -0.03 | -0.21, 0.16 | 0.7 | -0.07 | -0.28, 0.13 | 0.5 | -0.03 | -0.25, 0.19 | 0.8 |
| **EDSS^9^** | -0.20 | -1.7, 1.3 | 0.8 | -0.03 | -1.6, 1.6 | >0.9 | -2.0 | -3.7, -0.24 | 0.028 | -1.2 | -3.0, 0.64 | 0.2 |
| **PHQ8^2^ score** | 0.93 | 0.59, 1.3 | <0.001 |  |  |  |  |  |  |  |  |  |
| **FSMC^3^ score** |  |  |  | 0.22 | 0.13, 0.31 | <0.001 |  |  |  |  |  |  |
| **EQ5D^4^** |  |  |  |  |  |  | -16 | -23, -8.0 | <0.001 |  |  |  |
| **GSE^5^ score** |  |  |  |  |  |  |  |  |  | -0.46 | -0.74, -0.19 | 0.002 |

^1^BHADP: Barriers to Health Promoting Activities for Disabled Persons Scale

^2^PHQ8: Eight-item Patient Health Questionnaire Depression Scale

^3^FSMC: Fatigue Scale for Motor and Cognitive Functions

^4^EQ5D: EuroQol 5-Dimension 5-Level

^5^GSE: General Self-Efficacy Scale

^6^CI: Confidence Interval

^7^BMI: Body Mass Index

^8^MS: Multiple Sclerosis

^9^EDSS: Expanded Disability Status Scale

**Table S5. Sensitivity analysis – Imputed linear regression analyses with < or ≥ 7,000 steps/day as outcome.** Uni- and multivariable regression models to evaluate the association of dichotomized median step counts (< or ≥ 7,000 steps/day) assessed during the last week of the study with the BHADP scale assessed at the end of the study, based on the complete cases dataset (n=29) and imputed dataset (n=45).

| **Models** | **Univariate complete case analysis** | **Univariate imputed data analysis** | **Multivariate complete case analysis** | **Multivariable imputed data analysis^1^** |
| --- | --- | --- | --- | --- |
| ***Last week of the study*** |  |  |  |  |
| 1. **≥7,000 steps/d**^2^, odds ratio [95% CI^3^; *P*^4^] | 0.96  [0.82 to 1.11; .58] | 0.99  [0.88 to 1.1; .83] | 0.77  [0.48 to 1.06; .17] | 0.96  [0.83 to 1.12; .63] |
| 1. **≥7,000 steps/d**^2^ **controlled for steps/d**^2^ **at the end of the rehabilitation**, odds ratio [95% CI^3^; *P*^4^] | 0.76  [0.51 to 0.99; .09] | 0.9  [0.77 to 1.05; .18] |  | 0.83  [0.65 to 1.07; .14] |
| 1. **≥7,000 steps/d**^2^ **controlled for steps/d**^2^ **and barriers score at the end of the rehabilitation**, odds ratio [95% CI^3^; *P*^4^] | 0.77  [0.51 to 1; .11] | 0.91  [0.76 to 1.08; .25] |  | 0.85  [0.65 to 1.11; 1] |

^1^adjusted for age, sex, body mass index, multiple sclerosis duration, and Expanded Disability Status Scale

^2^*steps/day* corresponds to the mean number of steps per day and per individual

^3^95% CI: 95% confidence interval

^4^*P*: p-value

| **Models** | **Univariate complete cases analysis** | **Multivariable complete cases analysis^1^** |
| --- | --- | --- |
| ***Last week of the study*** |  |  |
| 1. **≥10,000 steps/d**^2^, odds ratio [95% CI^3^; *P*^4^] | 0.88 [0.69 to 1.05; .18] | 0.37 [NA to 0.8; .19] |
| 1. **≥10,000 steps/d**^2^ **controlled for steps/d**^2^ **at the end of the rehabilitation**, odds ratio [95% CI^3^; *P*^4^] | **0.72 [0.45 to 0.97; .09]** | 0 [0 to Inf; 1] |
| 1. **≥10,000 steps/d**^2^ **controlled for steps/d**^2^ **and barriers score at the end of the rehabilitation**, odds ratio [95% CI^3^; *P*^4^] | **0.71 [0.44 to 0.97; .10]** | 0 [0 to Inf; 1] |
| 1. **Steps/d**^2^, beta [95% CI^3^; *P*^4^] | -65.79 [-304.24 to 172.67; .58] | -92.15[-306.54 to 122.25; .38] |
| 1. **Steps/d**^2^ **controlled for steps/day**^2^ **at the end of the rehabilitation**, beta [95% CI^3^; *P*^4^] | **-219.51 [-413.43 to -25.59; .03]** | **-263.74 [-471.08 to -56.41; .02]** |
| 1. **Steps/d**^2^ **controlled for steps/day**^2^ **and barriers score at the end of the rehabilitation**, beta [95% CI^3^; *P*^4^] | -199.65 [-402.06 to 2.77; .05] | **-265.43 [-481.53 to -49.34; .02]** |
| 1. **≥150 min. of MVPA/wk**^5^ , odds ratio [95% CI^3^; *P*^4^] | 0.93 [0.8 to 1.07; .33] | 0.87 [0.68 to 1.08; .23] |
| 1. **≥150 min. of MVPA/wk**^5^ **controlled for min. of MVPA/wk**^4^ **at the end of the rehabilitation**, odds ratio [95% CI^3^; *P*^4^] | 0.85 [0.67 to 1.02; .11] | 0.79 [0.56 to 1.02; .11] |
| 1. **≥150 min. of MVPA/wk**^5^ **controlled for min. of MVPA/wk**^4^ **and barriers score at the end of the rehabilitation**, odds ratio [95% CI^3^; *P*^4^] | 0.84 [0.66 to 1.02; .10] | 0.76 [0.5 to 1.01; .10] |
| 1. **Min. of MVPA/wk**^5^, beta [95% CI^3^; *P*^4^] | -9.36 [-30.17 to 11.46; .36] | -11.65 [-34.3 to 11; .30] |
| 1. **Min. of MVPA/wk**^5^ **controlled for min. of MVPA/wk**^4^ **at the end of the rehabilitation**, beta [95% CI^3^; *P*^4^] | -15.98 [-34.21 to 2.24; .08] | -17.12 [-38.7 to 4.46; .11] |
| 1. **Min. of MVPA/wk**^5^ **controlled for min. of MVPA/wk**^4^ **and barriers score at the end of the rehabilitation**, beta [95% CI^3^; *P*^4^] | -13.25 [-32.41 to 5.92; .17] | -15.77 [-39.17 to 7.64; .18] |

**Table S6. Sensitivity analysis – Complete case linear regression analyses with PA as outcome.** Uni- and multivariable regression models to evaluate the association of PA, assessed during the last week of the study, with the BHADP scale, assessed at the end of the study, based on the complete cases dataset (n=29). Statistically significant effect sizes (p<0.05) are marked in bold.

^1^adjusted for age, sex, body mass index, multiple sclerosis duration, and Expanded Disability Status Scale.

^2^*steps/day* corresponds to the mean number of steps per day and per individual

^3^CI: Confidence interval

^4^*P*: p-value

^5^*min***.** *of MVPA/wk* corresponds to the sum of minutes of MVPA during the week

**Table S7. Sensitivity analysis – Linear regression analyses with PA assessed during the penultimate week of the study as outcome.** Uni- and multivariate regression models to evaluate the association of PA assessed during the penultimate week of the study with the BHADP scale, assessed at the end of the study, based on the complete cases dataset (n=29) and imputed dataset (n=45). Statistically significant effect sizes (p<0.05) are marked in bold.

| **Models** | **Univariate complete case analysis** | **Univariate imputed data analysis** | **Multivariate complete case analysis** | **Multivariate imputed data analysis** |
| --- | --- | --- | --- | --- |
| ***Penultimate week of the study*** |  |  |  |  |
| 1. **≥10,000 steps/d**^2^, odds ratio [CI^3^] | 1  [0.81 to 1.22; .99] | 0.99  [0.87 to 1.13; .89] | 0.94  [0.66 to 1.3; .69] | 0.98  [0.85 to 1.14; .83] |
| 1. **≥10,000 steps/d**^2^ **controlled for steps/d**^2^ **at the end of the rehabilitation**, odds ratio [CI^3^] | 0.68  [0.31 to 1.07; .21] | 0.89  [0.73 to 1.08; .24] |  | 0.8  [0.61 to 1.05; .10] |
| 1. **≥10,000 steps/d**^2^ **controlled for steps/d**^2^ **and barriers score at the end of the rehabilitation**, odds ratio [CI^3^] | 0.68  [0.31 to 1.07; .20] | 0.9  [0.72 to 1.11; .31] |  | 0.81  [0.59 to 1.1; .16] |
| 1. **Steps/d**^2^, beta [CI^3^] | -2.46 [-235.22 to 230.31; .98] | -9.38 [-211.57 to 192.81; .93] | -30.44 [-272.48 to 211.6; .80] | -29.5 [-238.78 to 179.78; .78] |
| 1. **Steps/d**^2^ **controlled for steps/day**^2^ **at the end of the rehabilitation**, beta [CI^3^] | -161.67 [-341.13 to 17.78; .08] | -127.04 [-268.73 to 14.66; .08] | **-253.68 [-466.34 to -41.02; .02]** | **-196.01 [-353.27 to -38.74; .02]** |
| 1. **Steps/d**^2^ **controlled for steps/d**^2^ **and barriers score at the end of the rehabilitation**, beta [CI] | -153.97 [-343.21 to 35.27; .11] | -117.84 [-259.83 to 24.15; .10] | **-257.85 [-479.24 to -36.46; .02]** | **-190.09 [-350.91 to -29.26; .02]** |
| 1. **≥150 min. of MVPA/wk**^4^ , odds ratio [CI^3^] | 1.02  [0.89 to 1.18; .77] | 1.01  [0.9 to 1.12; .91] | 0.97  [0.78 to 1.19; .76] | 1  [0.88 to 1.13; .96] |
| 1. **≥150 min. of MVPA/wk**^4^ **controlled for min. of MVPA/wk**^4^ **at the end of the rehabilitation**, odds ratio [CI^3^] | 0.97  [0.79 to 1.19; .79] | 0.99  [0.86 to 1.13; .86] | 0.87  [0.63 to 1.14; .34] | 0.96  [0.81 to 1.13; .58] |
| 1. **≥150 min. of MVPA/wk**^4^ **controlled for min. of MVPA/wk**^4^ **and barriers score at the end of the rehabilitation**, odds ratio [CI^3^] | 0.88  [0.66 to 1.11; .31] | 0.99  [0.86 to 1.14; .88] | 0.66  [0.3 to 1.02; .17] | 0.96  [0.81 to 1.13; .61] |
| 1. **Min. of MVPA/wk**^4^, beta [CI^3^] | -1.3 [-20.53 to 17.92; .89] | -3.02 [-17.32 to 11.29; .67] | -2.92 [-24.18 to 18.34; .78] | -4.11 [-19.35 to 11.13; .59] |
| 1. **Min. of MVPA/wk**^4^ **controlled for min. of MVPA/wk**^4^ **at the end of the rehabilitation**, beta [CI^3^] | -9 [-23.57 to 5.57; .22] | -6.15 [-17.76 to 5.46; .29] | -10.3 [-27.88 to 7.28; .24] | -7.52 [-20.94 to 5.89; .26] |
| 1. **Min. of MVPA/wk**^4^ **controlled for min. of MVPA/wk**^4^ **and barriers score at the end of the rehabilitation**, beta [CI^3^] | -8.88 [-24.49 to 6.73; .25] | -5.71 [-17.51 to 6.09; .33] | -10.67 [-29.79 to 8.45; .26] | -7.12 [-20.92 to 6.68; .30] |

^1^adjusted for age, sex, body mass index, multiple sclerosis duration, and Expanded Disability Status Scale

^2^*steps/d* corresponds to the mean number of steps per day and per individual

^3^CI: Confidence interval

^4^*min***.** *of MVPA/wk* corresponds to the sum of minutes of MVPA during the week
